# The South Asian Genome

**DOI:** 10.1371/journal.pone.0102645

**Published:** 2014-08-12

**Authors:** John C. Chambers, James Abbott, Weihua Zhang, Ernest Turro, William R. Scott, Sian-Tsung Tan, Uzma Afzal, Saima Afaq, Marie Loh, Benjamin Lehne, Paul O'Reilly, Kyle J. Gaulton, Richard D. Pearson, Xinzhong Li, Anita Lavery, Jana Vandrovcova, Mark N. Wass, Kathryn Miller, Joban Sehmi, Laticia Oozageer, Ishminder K. Kooner, Abtehale Al-Hussaini, Rebecca Mills, Jagvir Grewal, Vasileios Panoulas, Alexandra M. Lewin, Korrinne Northwood, Gurpreet S. Wander, Frank Geoghegan, Yingrui Li, Jun Wang, Timothy J. Aitman, Mark I. McCarthy, James Scott, Sarah Butcher, Paul Elliott, Jaspal S. Kooner

**Affiliations:** 1 Epidemiology and Biostatistics, Imperial College London, Norfolk Place, London, United Kingdom; 2 Imperial College Healthcare NHS Trust, London, United Kingdom; 3 MRC-HPA Centre for Environment and Health, Imperial College London, Norfolk Place, London, United Kingdom; 4 Ealing Hospital NHS Trust, Southall, Middlesex, United Kingdom; 5 Centre for Integrative Systems Biology and Bioinformatics, Imperial College London, London, United Kingdom; 6 Computational Biology and Statistics, University of Cambridge, Cambridge, United Kingdom; 7 NHLI, Imperial College London, Hammersmith Hospital, London, United Kingdom; 8 Wellcome Trust Centre for Human Genetics, University of Oxford, Oxford, United Kingdom; 9 Institute of Clinical Sciences, Imperial College London, London, United Kingdom; 10 Royal Brompton and Harefield Hospitals NHS Trust, London, United Kingdom; 11 MRC Clinical Sciences Centre, Imperial College London, London, United Kingdom; 12 Hero DMC Heart Institute, Dayanand Medical College and Hospital, Ludhiana, India; 13 BGI-Shenzhen, Shenzhen, China; 14 Oxford Centre for Diabetes, Endocrinology & Metabolism, University of Oxford, Churchill Hospital, Oxford, United Kingdom; 15 Oxford NIHR Biomedical Research Centre, Churchill Hospital, Oxford, United Kingdom; University of Alabama at Birmingham, United States of America

## Abstract

The genetic sequence variation of people from the Indian subcontinent who comprise one-quarter of the world's population, is not well described. We carried out whole genome sequencing of 168 South Asians, along with whole-exome sequencing of 147 South Asians to provide deeper characterisation of coding regions. We identify 12,962,155 autosomal sequence variants, including 2,946,861 new SNPs and 312,738 novel indels. This catalogue of SNPs and indels amongst South Asians provides the first comprehensive map of genetic variation in this major human population, and reveals evidence for selective pressures on genes involved in skin biology, metabolism, infection and immunity. Our results will accelerate the search for the genetic variants underlying susceptibility to disorders such as type-2 diabetes and cardiovascular disease which are highly prevalent amongst South Asians.

## Introduction

**Figure 1:**
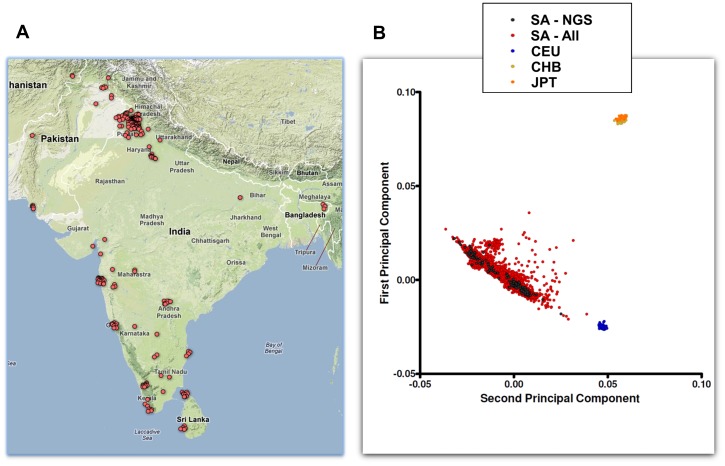
Location of birth (1A) and principal components analysis (PCA, 1B) of the South Asians sequenced. The PCA plots shows results for all South Asians in the LOLIPOP study (SA - All, red circles), for South Asians sequenced (SA - NGS, black dots) and for HapMap2 populations.

People originating from the Indian subcontinent (South Asians) comprise one-quarter of the globe's population. The 1000 Genomes Project has now systematically completed mapping the genomes of >1000 Africans, Americans, East Asians, and Europeans for genetic variation [1]. In contrast the genetic sequences of just two South Asians have been reported [2], [3]. South Asians are included in phases II and III of the 1000 Genome Project, but the present lack of knowledge of the South Asian genome remains an important obstacle to understanding the genetic mechanisms and biological pathways influencing the phenotypic differences and susceptibility to diseases among South Asians. We carried out next generation sequencing of 321 South Asians from different geographic regions, linguistic and religious groups (**Figure 1** **, Figure S1, Table S1 in File S1**) to compare genetic variation between people of South Asians ancestry and other major populations [4]–[6].

## Results

### DNA sequencing

We performed whole genome sequencing to a mean coverage of 4.3x (WGS-4x) amongst 168 South Asians. To provide detailed evaluation of coding regions, we then completed whole-exome sequencing (WES) amongst a further 147 South Asians. We report variant discovery based on these WGS-4x and WES data. We additionally carried out high-coverage WGS (WGS-28x, N = 8) to inform the accuracy of variant discovery and imputation in the low-coverage data. BAM files are available for download (European Nucleotide Archive: accession number PRJEB5476).

Sequencing was carried out using Illumina GAIIx and HiSeq2000 machines, with library preparation according to Illumina protocols. We performed sequence alignment and variant calling separately for WGS-4x, WGS-28x and WES. Short reads were aligned to reference genome (build 37) using BWA [7]. Sequencing metrics for WGS and WES are summarised in **Table S2 in File S1**. Mean coverage was 4.3x, 28.4x and 20.6x for WGS-4x, WGS-28x and WES respectively (**Figure S3**). Realignment around indels and variant calling were done using the Genome Analysis Toolkit (GATK) [8]. The quality scores for the initial set of SNPs and indels called were recalibrated, using dbSNP as a truth-set for machine learning, and filtered to provide SNP and indel call sets with predicted positive predictive values of 99.9% and 95% respectively. Mean TiTv ratio for SNPs was 2.12±0.01, 2.13±0.01 and 2.65±0.06 in WGS-4x, WGS-28x and WES respectively (**Figure S3**). The initial SNP Het/Hom ratio was 1.05±0.11 in WGS-4x, compared to 1.55±0.04 in WGS-28x, consistent with undercalling of heterozygotes in low-coverage sequencing. As expected, indels were enriched for variants that are multiples of 3 base-pairs long in coding regions (**Figure S4**) [9].

We next used BEAGLE [10] to simultaneously phase the WGS-4x data and, through haplotype inference, improve the accuracy of SNP and indel genotype calls. Het/Hom ratio for SNPs increased to 1.57±0.06 (**Figure S5**), and the proportion of SNPs with a difference of >1% in allele frequency compared to microarray data fell from 29% in the unphased WGS-4x data to 4.3% in the phased WGS-4x data (**Figure S6 and Figure S7**). After phasing and haplotype inference, concordance of SNP genotype calls with microarray data was comparable between the low coverage WGS-4x and the high coverage WES data (**Figure S8**). Positive predictive value for SNP calling was high for both phased WGS-4x and WES data, compared to results from genotyping by microarray (WGS-4x: 99.9±0.1%; WES: 99.7±0.1%, or direct genotyping of 252 known and novel SNPs (100% for both WGS-4x and WES, **Table S3 in File S2 and Figure S9**).

Compared to Illumina microarray, mean sensitivity for detection of SNPs was 91.9±0.2% and 99.2±0.1% in WGS-4x and WES respectively. Results were similar for comparisons of WGS-4x to WGS-28x SNP genotypes. We estimate that we have identified >95% of SNPs with non-reference allele frequency ≥0.5% in the mapped exome, and >95% of variants with non-reference allele frequency ≥1.0% in the remainder of the mapped genome (**Figure S10**).

We evaluated the accuracy of indel calling in the WGS-28x samples by Sanger sequencing. We confirmed the presence of an indel at 100% of 35 positions tested (**Table S4 in File S1**), enabling us to use our WGS-28x calls as a truth-set for the WGS-4x samples. Compared to indel genotypes called by WGS-28x, sensitivity and positive predictive value for indel genotypes called by WGS-4x were 58.4% and 96.0% respectively. Our findings are consistent with published data, and demonstrate the continued challenges posed by indel calling with low coverage data, even with state of the art approaches [11], [12].

### Genetic variation amongst South Asians

We identify 11,538,889 autosomal SNPs by WGS-4x and 189,939 by WES, with average 3,120,893 SNPs per person by WGS-4x and 34,698 by WES (**Table 1**, **Figure S5**). Overall we report 11,624,872 autosomal SNPs including 2,946,861 novel variants (**Table S5 in File S1**). We discovered 40,656 novel SNPs that are common (AF≥5%), 503,588 that are low frequency (AF≥1 and <5%) and 2,402,617 rare variants (AF<1%). In total we find 70,746 non-synonymous SNPs, 1,445 SNPs affecting stop codons, and 13,332 splice-site SNPs. Our discovery includes 30,914 novel nsSNPs, of which 2,960 are common or low frequency variants.

**Table 1:** SNPs and indels identified by low-coverage whole-genome sequencing (WGS-4x) and whole exome sequencing (WES) amongst South Asians.

	Per sample		All samples		
	WGS-4x	WES	WGS-4x	WES	WGS-4x & WES
SNPs	3,120,893	34,698	11,538,889	189,939	11,624,616
Novel SNPs	39,061	3,272	2,885,370	73,900	2,946,861
nsSNP	9,569	8,225	45,201	48,104	70,746
Novel ns SNPs	158	238	12,919	20,910	30,914
Indels	733,326	11,042	1,337,283	25,750	1,352,706
Novel indels	108,127	4,664	301,104	13,126	312,738
Inframe/frameshift indels	484	598	2,994	4,837	7,215
Novel inframe/frameshift indels	49	313	727	2,813	3,349

We report 1,337,283 autosomal indels by WGS-4x and 25,750 by WES, with an average 733,326 indels per person by WGS-4x and 10,217 by WES (**Table 1**). The novel autosomal indels include 237,326 common and 60,373 low frequency alleles. 7,215 of the indels are predicted to affect protein coding, of which 3,349 have not been previously described (**Table S6 in File S1**). In addition we report a further 370,719 SNPs and 43,121 indels on the sex chromosomes, of which 117,197 and 17,201 are newly described (**Table S5 and Table S6 in File S1**).

### Imputation of genotypes amongst South Asians

**Figure 2:**
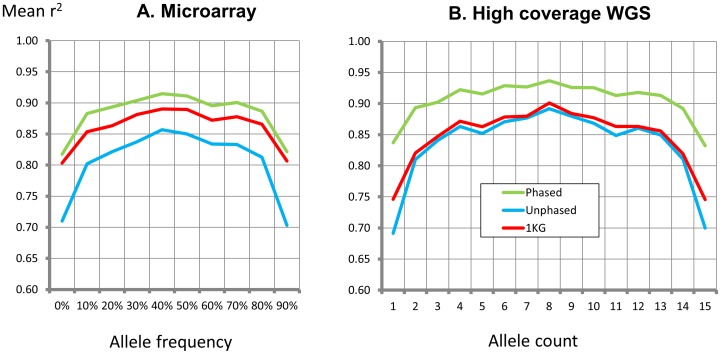
Correlation between imputed and observed genotypes amongst South Asians, using phased or unphased genotypes from low coverage WGS, or using 1000 Genomes Project data. Results are shown as mean r^2^ with genotypes observed from microarray data (**2A**) or high-coverage WGS (**2B**, WGS-28x).

We carried out imputation of the phased WGS-4x data amongst South Asians, quantifying accuracy by comparison to i. whole genome microarray data (Illumina 610, N = 6,557), and ii. high-coverage WGS-28x data (N = 8). Imputation accuracy for common variants was high using phased WGS-4x South Asian haplotypes, and better than imputation using either unphased South Asian data or haplotypes from the 1000 Genomes Project (**Figure 2**). Our South Asian haplotypes provide higher imputation accuracy than the 1000 Genomes Project dataset (**Figure S11**) even for low-frequency and rare variants.

The genotypes identified in our study thus provide the most accurate available whole genome reference panel for imputation of both shared and South Asian specific genetic variants. Our resource will enable genetic association studies to accurately identify variants underlying differences in phenotype and disease susceptibility between South Asians and other populations.

### Genetic variants shared with other populations and allelic stratification

We find that the genetic variants with AF>5% amongst South Asians are almost universally shared with all three of the 1000 Genomes populations (European [EUR], African [AFR] and East Asian [ASN]) confirming that most common genetic variants are cosmopolitan (**Figure S12**). However, allele frequencies show substantial heterogeneity between populations (**Figure S13**); this stratification may reflect founder effects, genetic drift or selective pressure. We used Wright's F_ST_ to identify alleles stratified between South Asian and the 1000 Genomes Project populations [13]. Mean F_ST_ between South Asians and European, East Asian and African populations was 0.010, 0.028 and 0.040 respectively; lower levels of F_ST_ between South Asians and Europeans are consistent with more recent admixture between these populations (**Figure S14**) [14].

**Figure 3:**
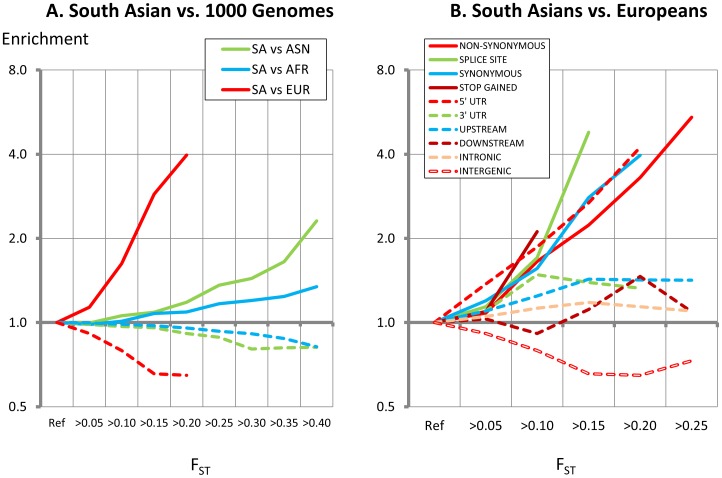
Enrichment for coding variants amongst autosomal SNPs stratified between South Asians and the 1000 Genome populations (3A) and for specific functional classes of SNPs amongst South Asians compared to Europeans (3B). Enrichment is calculated compared to null hypothesis; P values are provided in **Table S6 and Table S7 in File S1**.

We find that SNPs stratified between South Asians and Europeans are strongly enriched for variants in coding regions (P = 10^−4^ to 10^−15^, **Figure 3** **, Table S7 in File S1**). The SNPs stratified between South Asians and Europeans at F_ST_>0.10 are most enriched for non-synonymous, splice site, and 5′ UTR variants (1.4 to 2.1 fold enrichment; P = 10^−3^ to P = 10^−13^, **Figure 3** **, Table S8 in File S1**) with corresponding under-representation of intergenic SNPs (0.80 fold enrichment, P = 10^−135^). Our findings are consistent allelic stratification arising from selective pressure on functional genetic variants.

Allelic stratification shows regional clustering (**Figure S15**). Pathway analysis shows that the non-synonymous, splice-site, STOP and UTR SNPs most strongly differentiated between South Asians and Europeans (F_ST_>0.10, P<10^−13^) identify genes involved in the structure and function of the skin and eyes (*FLG*, *KRT3*, *HPS4*, *POU2F3*, *SLC45A2*, *TCHH*, *TYR*, and *TYRP1*), in metabolism (*AQP2*, *NEUROD1*, *PNPLA2*, *VDR* and *VLDLR*), and in infection and immunity (including *IFNGR1*, *ITGA4*, *ITGAE*, *ITGAL*, *SH2B3* and *TLR6*; **Table S9 in File S2 and Table S10 in File S1**, P<0.05).

**Figure 4:**
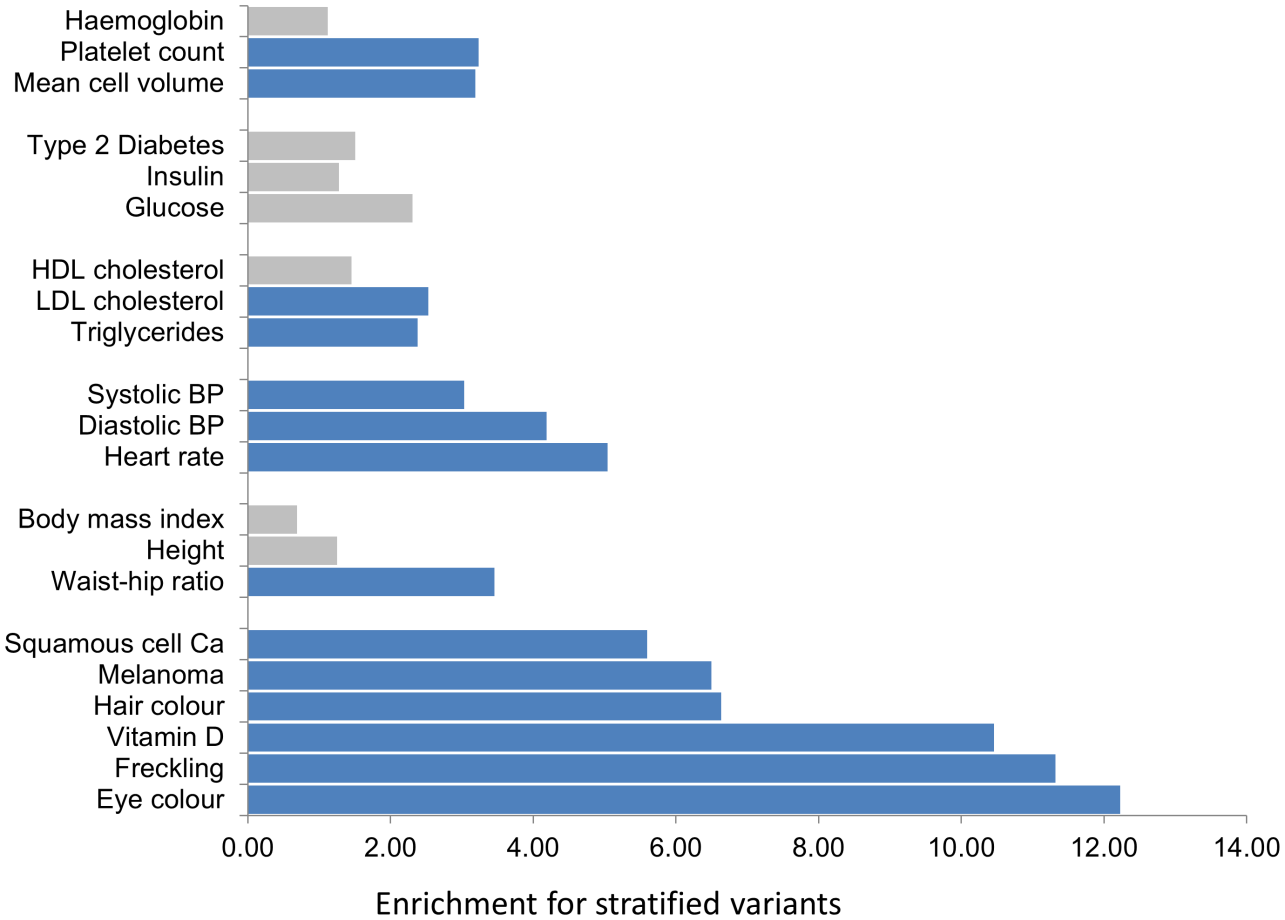
Enrichment for stratified genetic variants at genetic loci associated with respective phenotype in genome-wide association studies. Inset the correlation between the enrichment for stratified SNPs at known genetic loci, and enrichment of stratified variants for SNPs associated with respective phenotype in genome-wide association studies. Further details are provided in **Table S10 in File S1**.

We explored the potential biomedical relevance of allelic stratification. We find strong enrichment for differentiated alleles at genetic loci involved in susceptibility to skin cancers (6.4–10.8 fold enrichment, P<10^−3^, **Figure 4** **, Table S11 in File S1**). We also find allelic stratification at genetic loci associated with central obesity, blood pressure, fasting glucose and triglyceride levels, core components of the metabolic syndrome of insulin resistance (2.2–3.5 fold enrichment, P<0.05) that is 3–4 fold more prevalent common amongst South Asians than Europeans and considered to underlie their high cardiovascular disease risk [15], [16].

### South Asian specific genetic variation

We identify 2,946,861 novel SNPs (**Table S5**), including 40,656 variants that are common (AF≥5%), 503,588 that are low frequency (AF≥1 and <5%) and 2,402,617 rare variants (AF<1%). Novel genetic variants are less likely to be located in coding regions than expected under the null hypothesis, consistent with selection against sequence change in functional regions (P<10^−54^, **Figure S16, Table S12 in File S1**). As expected, selection is strongest against STOP and non-synonymous SNPs, and most evident at high allele frequencies (P = 10^−8^ to P = 10^−212^, **Table S12**). We find 568 coding-region SNPs specific to South Asians that are common (AF>5%). These are distributed between 502 genes; pathway analysis reveals these genes to be enriched for involvement in carbohydrate and lipid metabolism, including *IGF1*, *IGF2*, *LDLR* and *LYN* (P<0.01). Our results provide preliminary evidence for South Asian specific genetic variants in genes involved in energy storage and metabolism.

Most novel variants in South Asians are low-frequency or rare (>95%). Rare and low-frequency variants are responsible for familial disorders showing Mendelian patterns of inheritances, and may also contribute to the unexplained heritability of common disease [17]. Since rare and low frequency variants are not well captured by common haplotypes and are difficult to impute [18] (**Figure S11**), direct genotyping will be required for their evaluation; given the number of variants involved this will likely require a custom, South Asian specific microarray.

### Population structure among South Asians

Principal components analysis reveals a North-South gradient amongst South Asians, consistent with historic admixture of northern Indo-Aryan people (Ancestral North Indians, ANI) with predominantly southern Dravidian speakers (Ancestral South Indian, ASI). Sharing of genetic variants with European and African populations, is higher amongst South Asians with predominant ANI rather than ASI ancestry (P<10^−14^, **Figure S17**), whilst ASI have ∼30% more novel SNPs than ANI (P<10^−40^, **Figure S18 and Figure S19**). These observations are consistent with previous reports identifying ASI as the earliest inhabitants of the South subcontinent, and most diverged from other populations [14], [19].

## Discussion

South Asians have 2–4 fold higher risk of type-2 diabetes and cardiovascular disease compared to Europeans, but are protected from other conditions such as skin cancer [20]–[22]. The lack of knowledge of the patterns of genetic variation specific to South Asians has been a major limitation to identification of the genetic variants influencing disease susceptibility in this population [23].

Using whole-genome and whole-exome sequencing of South Asians we reveal 3 million new genetic variants, including 40,656 SNPs that are novel and common, and 503,588 that are novel low frequency SNPs. Population specific genetic variants can make an important contribution to disease. For example the 25 bp deletion in the gene encoding MYBPC3, present in ∼4% of people from the Indian subcontinent but not present in Europeans, confers an ∼7 fold risk of heart failure [24]. Our results will accelerate the search for South Asian specific genetic variation underlying the diseases of biomedical importance to this population. Our phased haplotypes will provide improved accuracy for imputation of genotypes in genome-wide association studies, and our catalogue of South Asian SNPs and indels will enable the design of custom microarrays to better study genetic variation in this population.

Skin cancer represents ∼20–30% of malignancies in Europeans, but just 1–2% of malignancies amongst South Asians. Epidermal melanin provides the primary mechanism for photoprotection against skin cancers [25]. We find evidence for allelic stratification between South Asians and Europeans of variants in *OCA2*, *SLC45A2*, *SLC24A4*, *TYR*, *TYRP* and other genes involved in melanin pigmentation of skin, hair and eyes [26]–[29]. The differentiation of risk alleles is sufficient to account entirely for the increased pigmentation amongst South Asians, and may explain the low incidence of skin cancers in this population [30], [31].

We find both population specific variants and allelic stratification in multiple genes implicated in energy conservation, including *APOH*, *IGF1* and *IGF2*, *LYN*, *LDLR*, *NEUROD1*, *PNPLA2* and *VLDLR*. APOH is a component of several circulating lipoproteins and is involved in activation of lipoprotein lipase [32]. Sequence variants in *APOH* are associated with LDL and triglyceride levels, and APOH plasma concentrations are elevated in people with T2D and metabolic syndrome [33]. *IGF1* and *IGF2* are growth factors which activate the insulin receptor [34], [35], whilst LYN is a tyrosine-kinase found in liver and adipose tissue which activates IRS1 leading to increased glucose utilisation [36], [37]. Our findings thus identify genetic variation amongst South Asians involving a cluster of genes linked to core metabolic traits including lipid metabolism, adipogenesis, insulin signalling and T2D. This pattern of genetic may have been advantageous amongst South Asians in historic conditions, consistent with a in the context of unstable food supplies [38]. These potentially protective variants may have become deleterious in changing environments such as from rural to urban, and to westernised societies, and may contribute to the increased risk of T2D and obesity after migration amongst South Asians.

We identify stratification on many genes involved in immune cell biology including *IFNGR1*, *ITGA4*, *ITGAE*, *ITGAL*, *SH2B3* and *TLR6*. In particular, *IFNGR1* encodes a component of the interferon-gamma receptor, a key signalling mechanism in immune activation. SNP rs1887415 in *IFNGR1* is ∼20-fold more common amongst South Asians than Europeans; it introduces an amino acid change in a domain reported to be involved in STAT signalling, a major regulator of inflammation [39]. Genetic variants in *IFNGR1* influence susceptibility to mycobacterial infection and cerebral malaria [40], [41], major infectious diseases which are endemic on the South subcontinent.

Our discovery of the genetic variants that are specific to South Asians will now enable the design of customised microarrays to search for the DNA sequence variants underlying susceptibility to the type-2 diabetes and cardiovascular disease, which are highly prevalent amongst South Asians. Our results also provide a genome wide survey of allelic stratification between South Asians and other populations. This resource will form the basis of future studies to investigate the ancestral origins and selective pressures that have shaped this major population group.

## Methods

### Participants

We sequenced 321 unrelated men of self-reported South Asian ancestry (all 4 grandparents born on the Indian subcontinent), participating in the London Life Sciences Population (LOLIPOP) study and living in the UK [42]. LOLIPOP is a representative sample of UK South Asians. Samples were selected based on the following criteria: i. GWA data available to enable assessment for population stratification and genotype concordance; ii first generation migrant (ie born on the Indian subcontinent) and able to provide the town nearest to their place of birth. The samples selected for 4x-WGS were all male, to provide equal coverage of the X and Y chromosomes. Sampling was otherwise at random. The samples sequenced included individuals from a range of geographic regions, religious and linguistic subgroups (**Table S1 in File S1**). Principal components analysis using whole genome SNP data (Illumina Hap610 microarray) available for participants confirmed that the South Asians sequenced were representative of South Asians living on the Indian subcontinent (**Figure S1 and Figure S2**). The research was approved by the West London Research Ethics Committee (reference number: 07/H0712/150); all participants gave written informed consent.

### Library preparation

DNA libraries were prepared according to Illumina instructions. For WGS, 3–5 µg of genomic DNA were fragmented by nebulization with compressed nitrogen gas at 35 p.s.i. for 6 min. DNA fragments with overhangs were end-repaired using T4 and Klenow polymerases and T4 polynucleotide kinase with 10 mM dNTP's followed by addition of an “A” base at the ends using Klenow exo fragment (3′ to 5′-exo minus) and dATP (1 mM). Sequencing adaptors containing “T” overhangs were ligated to the DNA products followed by agarose (2%) gel electrophoresis. Fragments of about 400 bp were isolated from the gels (Qiagen Gel Extraction Kit) and the adaptor-modified DNA fragments were PCR enriched for 10-cycles using Phusion DNA polymerase (Finnzymes Oy) and primers PE 1.0 and PE 2.0 (Illumina). Enriched libraries were further purified using agarose (2%) gel electrophoresis as described above. The quality and concentration of the libraries was assessed using Nanodrop absorbance and by the Agilent 2100 Bioanalyzer using the DNA 1000 LabChip (Agilent).

For WES, 1 mcg of DNA was sheared using the Covaris E-Series, and libraries prepared according to the Illumina TruSeq method manual, using a gel-free protocol. Samples were tagged using Illumina adapters before pooling. Samples were quantified using the Invitrogen Qubit QuantIT system and the Agilent Bioanalyzer HS DNA chip to check library sizing. Libraries were then further quantified using the Kapa SYBR Fast Library Quant Kit for Illumina GA (Anachem) to more accurately quantify adapter-ligated DNA present in each sample library. Sample libraries were pooled in groups of 6, all with differing tags, at 500 ng/sample, for a total DNA library mass of 3000 ng per pool. Exome capture was performed using the Illumina TruSeq Exome Enrichment kit, according to the provided method manual. Post-enrichment, samples were again quantified using Qubit and Bioanalyzer, and then again with the Kappa Library Quant Kit. Samples were diluted to 2 nM in preparation for sequencing.

### DNA sequencing

Paired-end sequencing-by-synthesis was performed on Illumina GAII_x_ instruments (WGS) or HiSeq 2000 (WES). Template DNA fragments were hybridized to the surface of the Illumina flow cells and amplified to form clusters using the Illumina cluster station. In brief, DNA (8–10 pM) was denatured followed by hybridization to grafted adaptors on the flow cell. Isothermal bridge amplification using Phusion polymerase was then followed by linearization of the bridged DNA, denaturation and blocking of 3′-ends and hybridization of the sequencing primer. Samples were sequenced using 2×101 cycles (WGS) or 2×100 cycles (WES) of incorporation and imaging. Imaging and analysis of the data was performed using the SCS 2.6 and RTA 1.6 software packages from Illumina, respectively. RTA analysis involved conversion of image data to base-calling in real-time.

### Sequence alignment

Default parameters were used for all data processing and analysis stages unless otherwise specified. Each sample was assessed for sequence quality on the basis of per-base and per-sequence quality scores, GC content, duplication levels, kmer content and sequence over-representation using FastQC version 0.10.0. Low-quality sequence was trimmed from reads using the fastq_quality_trimmer component of the fastx toolkit version 0.0.13, removing bases from the 3′ end of reads with a quality score less than 15, and discarding reads which were shorter than 60 bp following quality trimming. Reads were also processed with cutadapt to remove any residual sequencing adapters. Duplicate reads resulting from both PCR and optical duplicates were identified using Picard's MarkDuplicates tool, using an optical pixel duplicate parameter of 100.

Sequence reads were aligned to build 37 patch 5 of the human genome sequence using bwa [7] version 0.6.1. Suffix array co-ordinates of individual reads were determined with the ‘aln’ method, using unseeded alignments with gap opening and extension penalties of 11 and 4 respectively, with 1 gap allowed per alignment. SAM format alignments were generated using the ‘sampe’ method, reporting a single alignment for each read-pair, and written as BAM formatted files by filtering the SAM format alignments through SAMtools [43] version 0.1.9. BAM files are available for download (European Nucleotide Archive: accession number PRJEB5476).

Alignments were then sorted by co-ordinate and a BAM index generated using SAMtools. A read-group header (RG) was added to each bam file using Picard to allow differentiation of samples amongst pooled data sets. Individual alignments were then merged into pooled BAM format files. The alignments from the low-coverage genomes were then split into individual chromosomes, whereas the exome sequence alignments were left in a single merged file.

Local re-alignments were carried out around indels in the aligned reads using the GATK [8]. Sites requiring realignment were identified with GATK's RealignerTargetCreator, with known indel loci provided from 1000 Genomes Phase I Indels and the ‘Mills-Devine Gold Standard’ Indels (http://genome.cshlp.org/content/21/6/830.long). The identified target loci were passed to the GATK IndelRealigner, with realignments carried out using the Smith-Waterman consensus determination model. The LOD threshold for cleaning was reduced to 3.0 in the case of the low-coverage genome samples, but left at the default of 5.0 for exomic or high-coverage sequences. Finally the GATK CountCovariates tool was used to assess a number of covariates (ReadGroupCovariate, QualityScoreCovariate, CycleCovariate and dinucleotideCovariate), with known variant loci provided from dbSNP. The resulting recalibration data was applied to correct base quality scores using the TableRecalibration tool.

### Variant calling, filtration and phasing

The GATK Unified Genotyper was used for the identification of both SNPs and Indels, using dbSNP 135 for identification of known variant loci. The Unified Genotyper was run using ‘discovery’ genotyping mode (reporting the most likely alternate allele), and alternately using ‘SNP’ and ‘INDEL’ genotype likelihood models. In the case of SNP identification, the default minimum confidence threshold for calling (30) was applied with exome sequences, but this was reduced to 4 for the low coverage genomes. Exome sequences and high-coverage genomes were analysed using a coverage threshold of 200, beyond which down-sampling was applied to the reads, whereas low-coverage genomes had a coverage threshold of 50 applied.

Filtering of SNP calls was carried out using the GATK VariantRecalibrator using HapMap 3.3 as both truth and training sets (prior = 15), the 1000 Genomes genotypes from the Omni 2.5 chip as a training set (prior = 12) and dbSNP 135 for the known data set (prior = 8). Annotations used in the Gaussian mixture were DP (Depth of reads passing quality thresholds), QD (Quality by depth), Haplotype Score, Mapping quality rank sum test, read position rank sum test and Inbreeding coefficient. Only variants falling into the >99.9% truth-sensitivity tranche were retained for analysis. Recalibration of genomic indels was carried out using a similar process, but omitting the QD annotation from the recalibration process, and using the Mills-Devine/1000G Indel set for truth and training sets. Indel calls derived from exome sequencing were manually filtered using thresholds of QD<2.0, ReadPosRankSum<40.0, FS>200, InbreedingCoeffecient<−0.8. Despite variant recalibration, a high proportion of indel calls in the low-coverage WGS were false positive findings; we therefore only retained low-coverage indel calls at sites that were also identified as polymorphic in the high-coverage data.

Unphased likelihoods from variants called from low-coverage genomes were prepared using the GATKs ProduceBeagleInput tool, and haplotype inference carried out using Beagle version 3.3.2 [10], with 50 phasing iterations. The resulting phased genotypes were incorporated with the vcf-format variants using GATK's BeagleOutputToVCF utility. SNPs were annotated using the NGS-SNP [44] package while indel annotation was carried out using ANNOVAR [45]. In both cases annotation was carried out against an Ensembl v67 reference database. SNPs and indels were considered novel if not present in dbSNP 135.

### Accuracy of variant calling and imputation

We assessed the accuracy of SNP calling by the WGS-4x and WES data using three datasets: i. 537K SNP genotypes measured by the Illumina 610 microarray and ii. 252 SNPs identified by WGS or WES submitted to replication genotyping by KASPar, and iii. results from two samples analysed by both WGS-4x and WGS-28x. The 252 SNPs submitted for direct genotyping were specifically selected to be non-synonymous variants that were either i. novel (N = 103) or ii. known but showing evidence for stratification between South Asian and Europeans (F_ST_>0.15, N = 149). Direct genotyping was done amongst up to 2,638 South Asians. Our strategy thus provides a robust assessment of calling error rate and allele frequency estimates in conserved regions identified as showing the greatest evidence for departure from expectation. We defined sensitivity as the proportion of sites polymorphic in the reference set that were identified as polymorphic in the experimental (WGS-4x or WES) set. Positive predictive value was defined as the proportion of sites identified as polymorphic in the experimental (WGS-4x or WES) call set that were confirmed to be polymorphic in the reference set.

We validated 35 indels using Sanger sequencing. The indel set comprised 18 known indels (Broad Institute set of Mills Devine and Thousand Genome indels) and 17 novel previously undescribed variants. Validation was carried out amongst eight individuals predicted to carry the indels by WGS-28x. PCR primers were designed using BatchPrimer3 (v1.0) or the Primer3Plus2 software (**Table S13 in File S1**). PCR was carried out with reaction volumes of 25 µl on a Bio Rad Peltier Thermal Cycler with BIOTAQ DNA Polymerase (Bioline). PCR products were purified for sequencing using MultiScreen PCR_μ96_ filtration plates (Millipore) and Sanger Sequenced on the ABI 3730 xl.

We carried out imputation amongst 6,557 South Asians previously genotyped using the Illumina 610 microarray and not included in the discovery experiment; this included the 8 South Asians with WGS-28x data. Imputation was done for chromosome 22 using IMPUTE2 [46], with either phased WGS-4x or 1000 Genomes Project genotypes as the reference set. Concordance was quantified as the correlation (r^2^) between predicted and observed genotypes doses, and was assessed separately in comparison to observed microarray and WGS-28x genotypes. Correlations were calculated for each genomic site predicted to be polymorphic by imputation, and results averaged by allele frequency (microarray) or allele count (WGS-28x).

### Population stratification and pathway analysis

To identify regions of the genome most closely associated with South Asian ancestry, we determined values for Wright's F_ST_ as a measure of population differentiation that may arise from founder effects, genetic drift or through selective pressure [13]. We carried out pairwise comparisons of allele frequencies in South Asians (determined by WGS-4x), with those reported amongst African, East Asian and European populations (1000 Genomes Project). In each pairwise comparison, analysis was limited to SNPs present in both populations. We tested whether SNPs with high F_ST_ were enriched for specific functional classes of variant, compared to distribution expected under the null hypothesis (χ^2^ test). We carried out pathway analysis for genes containing SNPs with high F_ST_ using Ingenuity Pathway Analysis [47]. For the enrichment analyses of stratified variants, expectations under the null hypothesis were generated by permutation testing. For each phenotype, we generated 10,000 random sets of sentinel SNPs matched to the published genome-wide association sentinel SNPs based on allele frequency (+/−0.02), gene proximity (+/−10 kb) and number of genes within 250 kb (+/−2), and counted the number of stratified SNPs falling within 500 kb of the random sentinel SNP set. Similar results were obtained using a 1 MB window.

### Reference datasets

Where applicable, data sets for the analysis of the sequences were those distributed with version 1.5 of the Genome Analysis Toolkit (GATK) resource bundle. These include the Human Genome Reference Consortium build 37 patch 5 release of the Human genome, including unlocalised contigs and the rCRS MT sequence, dbSNP build 135, HapMap 3.3 and the Broad Institute curated Mills-Devine/1000 Genomes indel set. Comparison with variants identified by the 1000 Genomes project was made against the April 2012 Integrated Variant Set.

## Supporting Information

Figure S1 **Principal components analysis of samples sequenced by WES or WGS-4x, compared to HapMap 2 (1A &1B) and HapMap 3 (1C and 1D) samples.** Results are shown for all South Asians in the LOLIPOP study (SA-All, red circles), and for South Asians sequenced (IA-NGS, black dots).
(TIF)

Figure S2 **Principal component analysis of genotype data for i. UK South Asian samples sequenced by WES or WGS in the current study, ii. HapMap CEU samples, iii. HapMap CHB/JPT samples, and iv. Indian samples studied by Reich et al (Nature 2009).** The UK South Asian samples studied coincide with the Reich samples collected in India, confirming they are representative of non-migrant South Asians. The Reich samples include Aonaga and Nyishi peoples, small South Asian subpopulations from the north-east region of India, with strong East Asian influence.
(TIF)

Figure S3 **Principal component analysis of genotype data for South Asian samples from the LOLIPOP study.** Samples selected for WES or WGS in the current study are identified by red symbols.
(TIF)

Figure S4 **Sequencing statistics for the WGS-4x (red) and WES (blue) samples.**
(TIF)

Figure S5 **Enrichment of indels for variants multiples of 3 bases long in coding regions.**
(TIF)

Figure S6 **SNP call metrics in unphased (red) and phased (blue) low-coverage WGS-4x data.**
(TIF)

Figure S7 **Allele frequencies in unphased (left panel) and phased (right panel) WGS-4x data compared to results from direct genotyping by microarray.**
(TIF)

Figure S8 **Difference in allele frequency between WGS-4x and microarray data, before and after phasing.**
(TIF)

Figure S9 **Concordance of genotype calls from unphased WGS-4x, phased WGS-4x or WES, with calls from microarray.**
(TIF)

Figure S10 **Allele frequency for 252 known and novel variants identified by WGS-4x or WES, compared to results from single variant genotyping in separate sample of up to 2,638 South Asians.**
(TIF)

Figure S11 **Power for discovery of SNPs at differing allele frequency by WGS-4x, by WES or in combination.** Calculations are based on observed sensitivity for SNP detention of 91.9% by WGS-4x (N = 168) and 99.2% by WES (N = 147).
(TIF)

Figure S12 **Proportion of autosomal SNPs identified by WGS-4x that are in low LD (r^2^<0.5) with tag-SNPs on the llumina 610 microarray.**
(TIF)

Figure S13 **Sharing of South Asian SNPs identified by WGS-4x with 1000Genomes populations.**
(TIF)

Figure S14 **Allele frequencies for autosomal SNPs identified by WGS-4x amongst South Asians (x-axis), compared to 1000 Genomes populations (y-axis).** Results shown only for SNPs shared between the respective populations.
(TIF)

Figure S15 **Distribution of F_ST_ values between South Asians and the 1000 Genomes populations for autosomal SNPs identified by WGS-4x.**
(TIF)

Figure S16 **Manhattan plot showing F_ST_ values for autosomal coding SNPs amongst South Asians compared to Europeans on chromosomes 4–6.** Regional plots are shown for SNPs with F_ST_>0.30. In the regional plots the SNP with highest F_ST_ are identified by blue diamonds, while other SNPs are colour coded according to their LD with the sentinel (r^2^>0.8: red; r^2^>0.5 and ≤0.8: orange; r^2^>0.2 and ≤0.5: yellow).
(TIF)

Figure S17 **Enrichment for SNP functional classes across a range of allele frequencies for South Asian specific SNPs (A) and for SNPs shared with other populations (3B).** Enrichment is calculated compared to null hypothesis; P values are provided in Table S12.
(TIF)

Figure S18 **Sharing of autosomal SNPs identified by WGS-4x amongst ANI and ASI with 1000Genomes populations.**
(TIF)

Figure S19 **Novel SNPs and Het-Hom ratio amongst ANI and ASI South Asians.**
(TIF)

Figure S20 **Relationship between ANI-ASI gradient and the number of novel autosomal SNPs identified by WGS-4x.** ASI-ANI gradient quantified by principal components analysis (x axis) where lower PC indicates greater ASI content.
(TIF)

File S1 **Table S1, Characteristics of participants.** **Table S2, Per sample sequencing metrics for WGS and WES.** Results are mean (SD). **Table S4, Results of indel validation by Sanger sequencing.** Sanger sequencing of 35 indel (17 previously unreported) amongst 8 individuals predicted to carry the indels by WGS-28x. Sanger sequencing confirmed the presence of indels at all sites called by whole genome sequencing. For 33 of the 35 indels (94.3%) Sanger sequencing showed identical sequence to that predicted by WGS-28x. Sanger sequencing of the remaining two PCR amplicons confirmed presence of a complex indel within 20 nucleotides of the originally called indel; one fell within a repetitive region and the second was difficult to analyse. Both these indels were novel. **Table S5, Functional class of SNPs identified by WGS-4x and WES. Table S6, Functional class of indels identified by WGS-4x and WES. Table S7, P values for enrichment of autosomal coding and intergenic SNPs across the range of F_ST_ between South Asians and the AFR, ASN or EUR populations. Table S8, P values for enrichment of functional classes amongst autosomal SNPs across the range of F_ST_ between South Asians and 1000 Genomes Project populations. Table S10, Pathway analysis (Ingenuity Pathway Analysis) of genes with potentially functional SNPs stratified between South Asians and Europeans (F_ST_>0.10). Table S11, Enrichment for stratified SNPs at genetic loci known to be associated with respective phenotype in GWA studies.** Observed: no of stratified SNPs (Fst>0.10) within 500 kb of the reported sentinel SNPs. Predicted: mean no of SNPs expected to fall within 500 kb of the sentinel SNPs under null hypothesis. Expectation based on permutation testing: 10,000 runs of SNP sets matched to the stratified SNPs based on allele frequency and gene proximity, but otherwise selected at random. Enrichment: observed/expected. P: exact probability of the observed based on the distribution of expected generated by permutation testing. **Table S12, P values for enrichment of functional classes amongst autosomal SNPs across a range of allele frequencies.** **Table S13, PCR primers for validation of indel calling by Sanger sequencing.**
(DOCX)

File S2 **Table S3, Replication results for 252 SNPs genotyped by single variant tests amongst up to 2,638 South Asians.** Dataset: sequence data SNP was selected from. AF: allele frequency. **Table S9, Coding, splice and UTR SNPs showing greatest stratification between South Asians and Europeans.** AF: allele frequency, SA: South Asians, AFR; Africans, ASN: East Asians; EUR: Europeans.
(XLSX)
