# Supplementary figures and images for: The South Asian Genome
Source: PLoS One. 2014 Aug 12;9(8):e102645. doi: 10.1371/journal.pone.0102645 (PMC4130493; doi:10.1371/journal.pone.0102645)

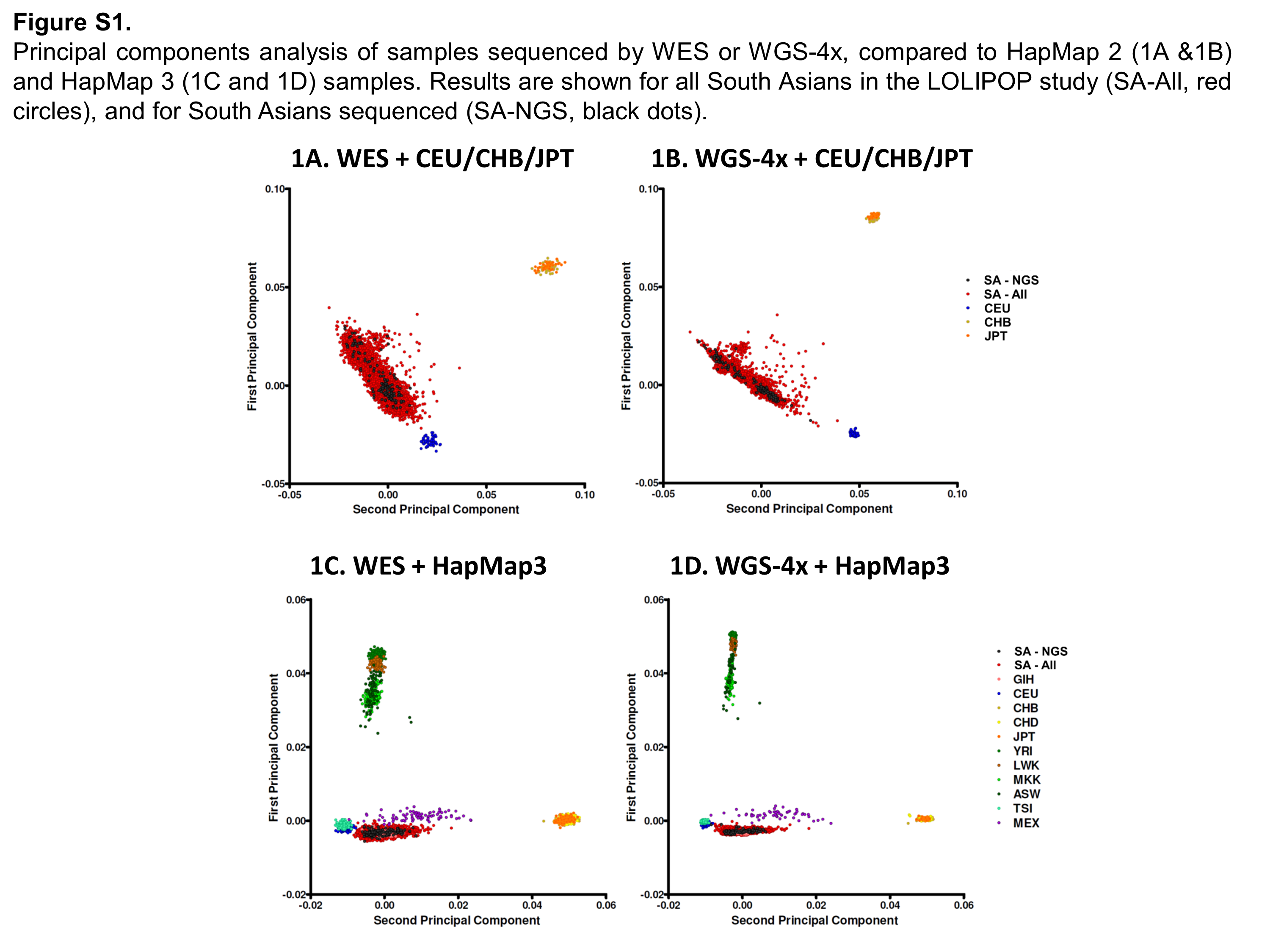

Supplement: Figure S1 — Principal components analysis of samples sequenced by WES or WGS-4x, compared to HapMap 2 (1A &1B) and HapMap 3 (1C and 1D) samples. Results are shown for all South Asians in the LOLIPOP study (SA-All, red circles), and for South Asians sequenced (IA-NGS, black dots). (TIF) [file pone.0102645.s001.tif]

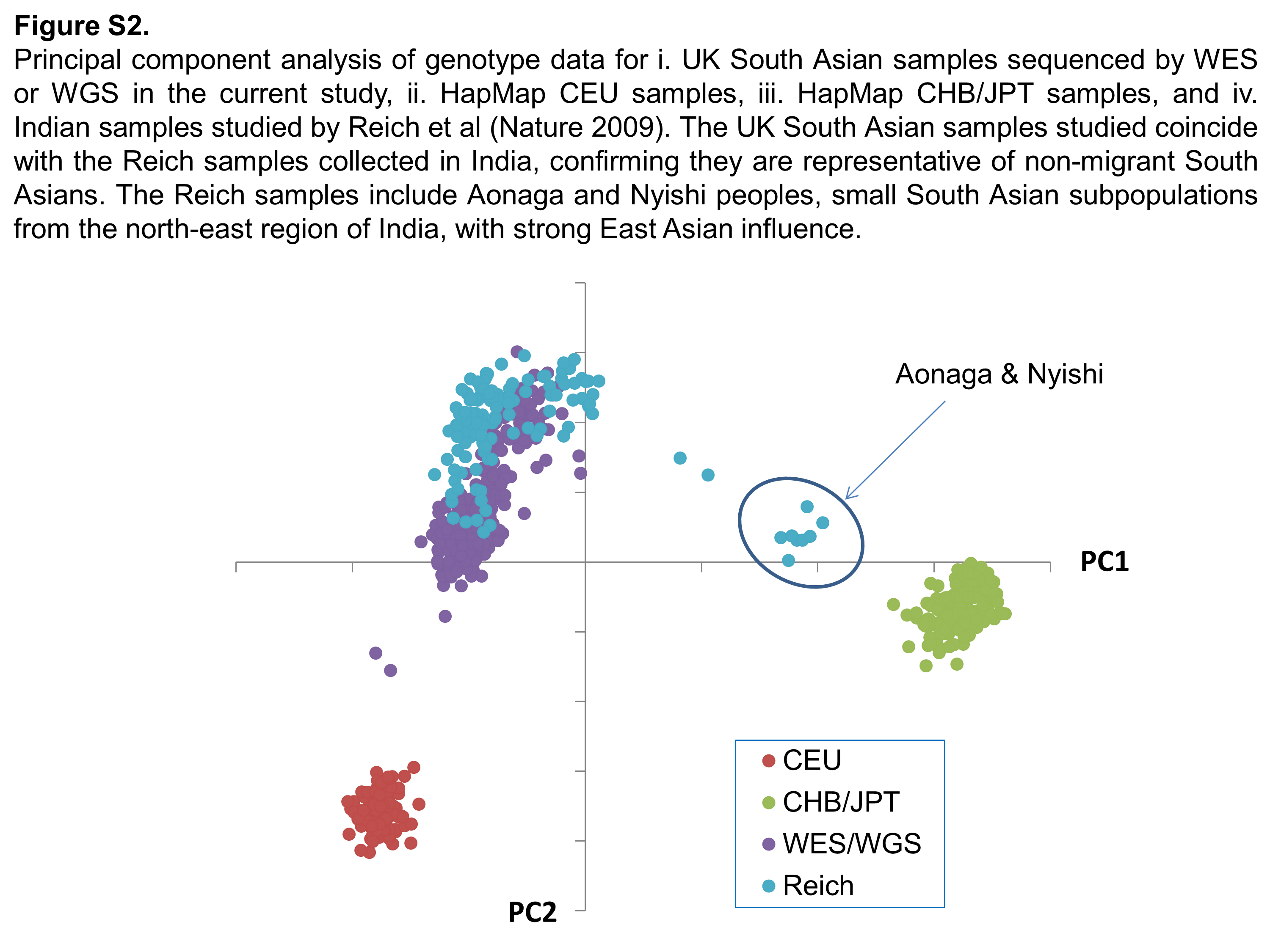

Supplement: Figure S2 — Principal component analysis of genotype data for i. UK South Asian samples sequenced by WES or WGS in the current study, ii. HapMap CEU samples, iii. HapMap CHB/JPT samples, and iv. Indian samples studied by Reich et al (Nature 2009). The UK South Asian samples studied coincide with the Reich samples collected in India, confirming they are representative of non-migrant South Asians. The Reich samples include Aonaga and Nyishi peoples, small South Asian subpopulations from the north-east region of India, with strong East Asian influence. (TIF) [file pone.0102645.s002.tif]

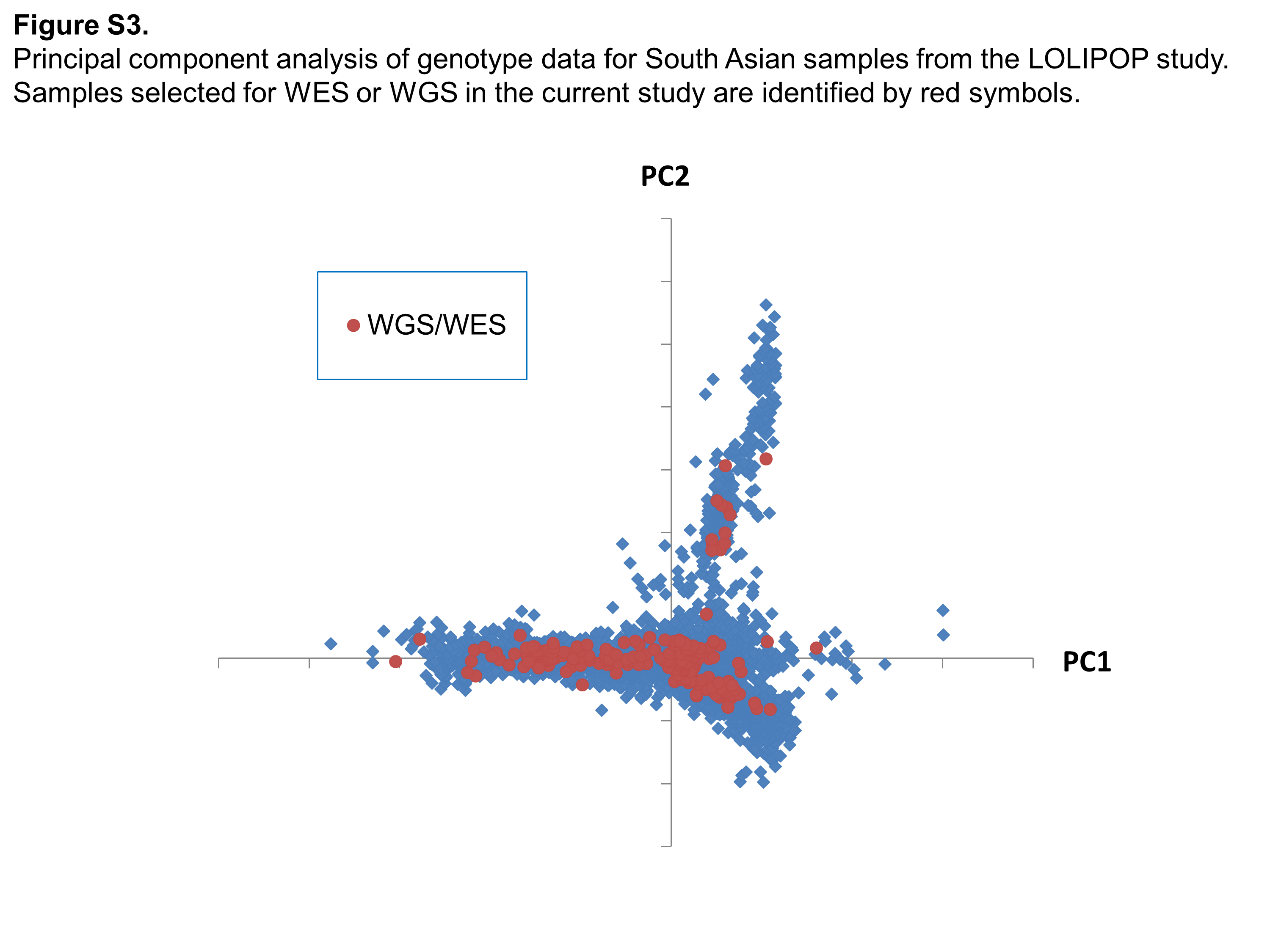

Supplement: Figure S3 — Principal component analysis of genotype data for South Asian samples from the LOLIPOP study. Samples selected for WES or WGS in the current study are identified by red symbols. (TIF) [file pone.0102645.s003.tif]

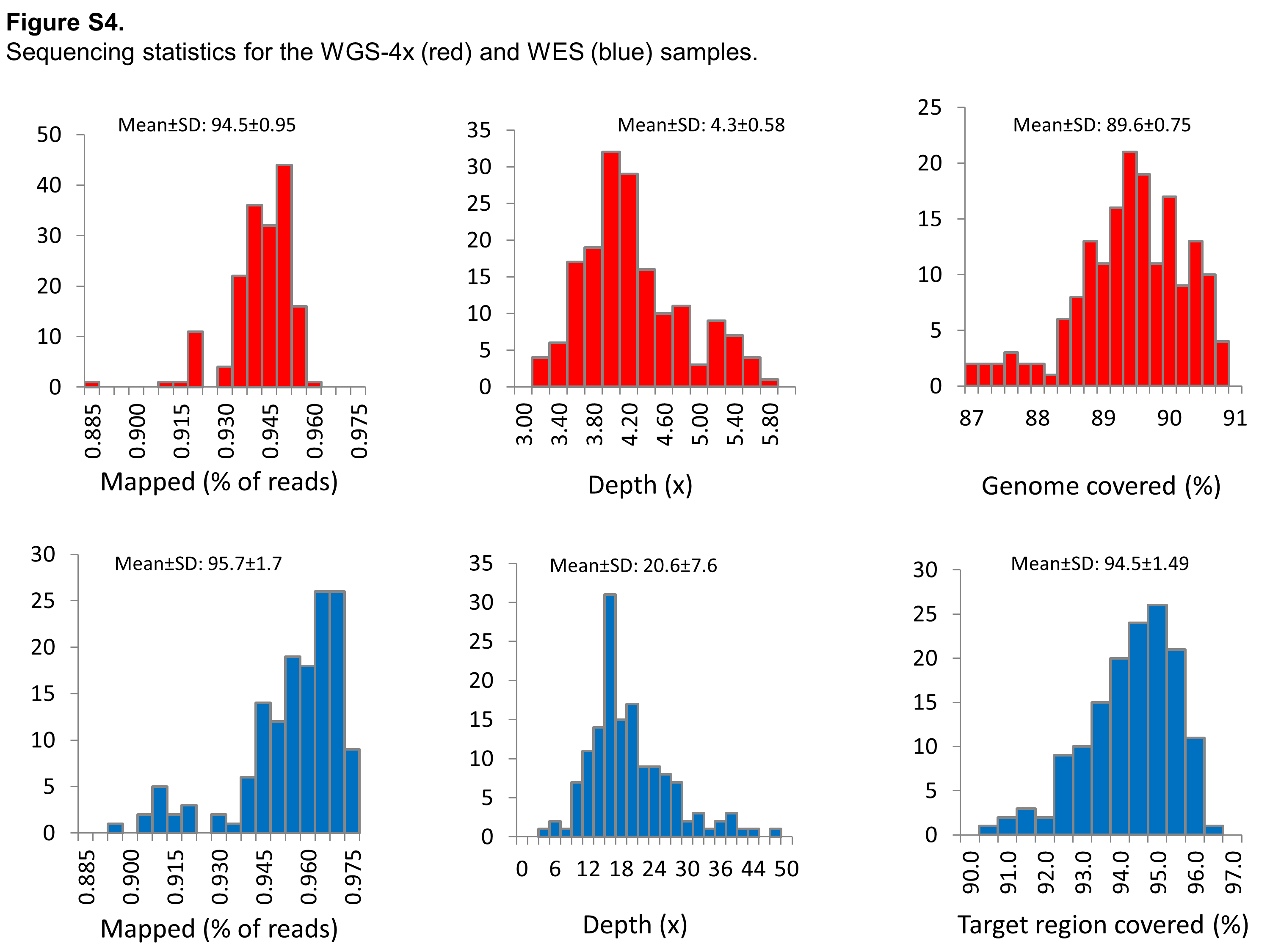

Supplement: Figure S4 — Sequencing statistics for the WGS-4x (red) and WES (blue) samples. (TIF) [file pone.0102645.s004.tif]

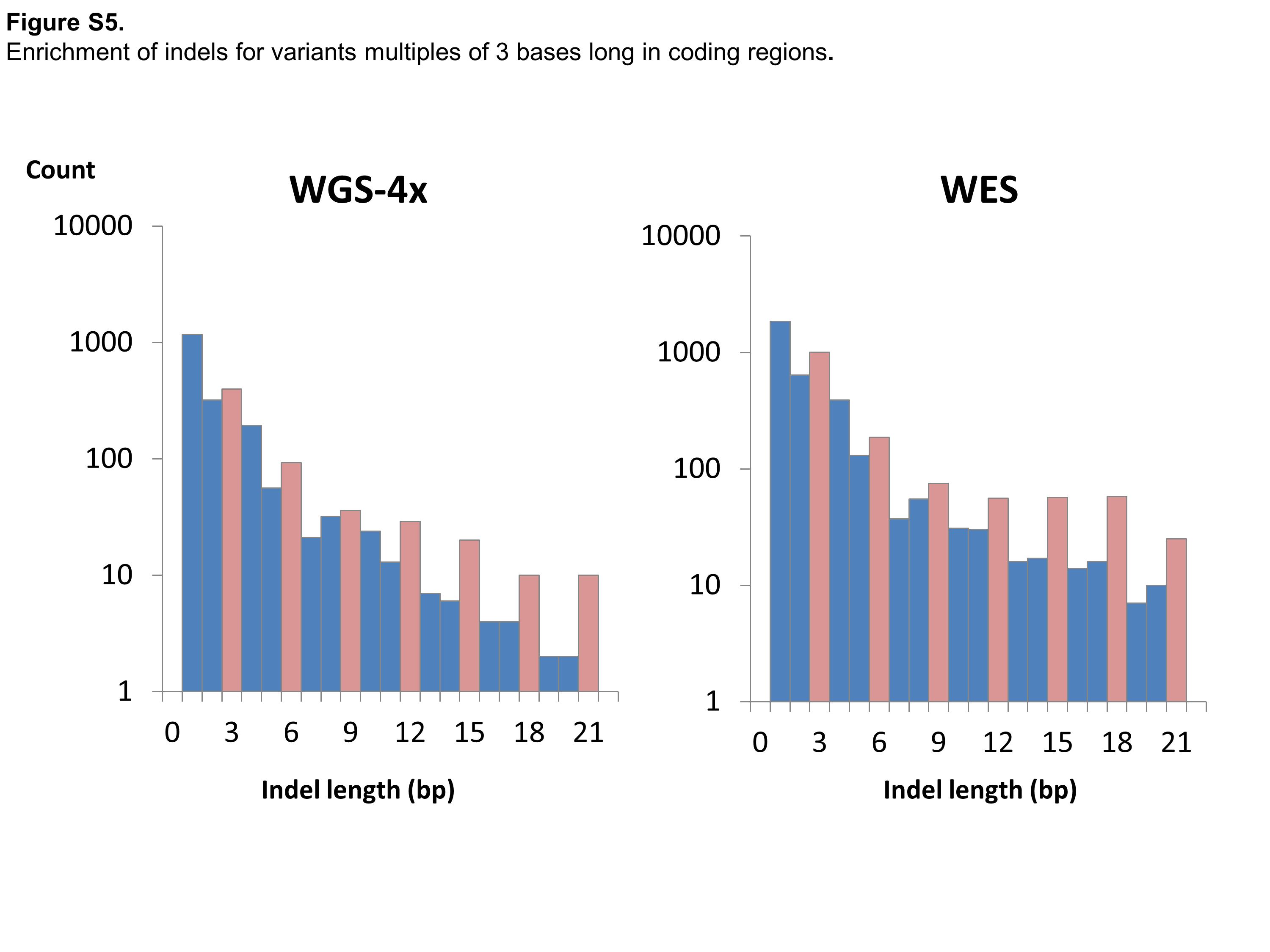

Supplement: Figure S5 — Enrichment of indels for variants multiples of 3 bases long in coding regions. (TIF) [file pone.0102645.s005.tif]

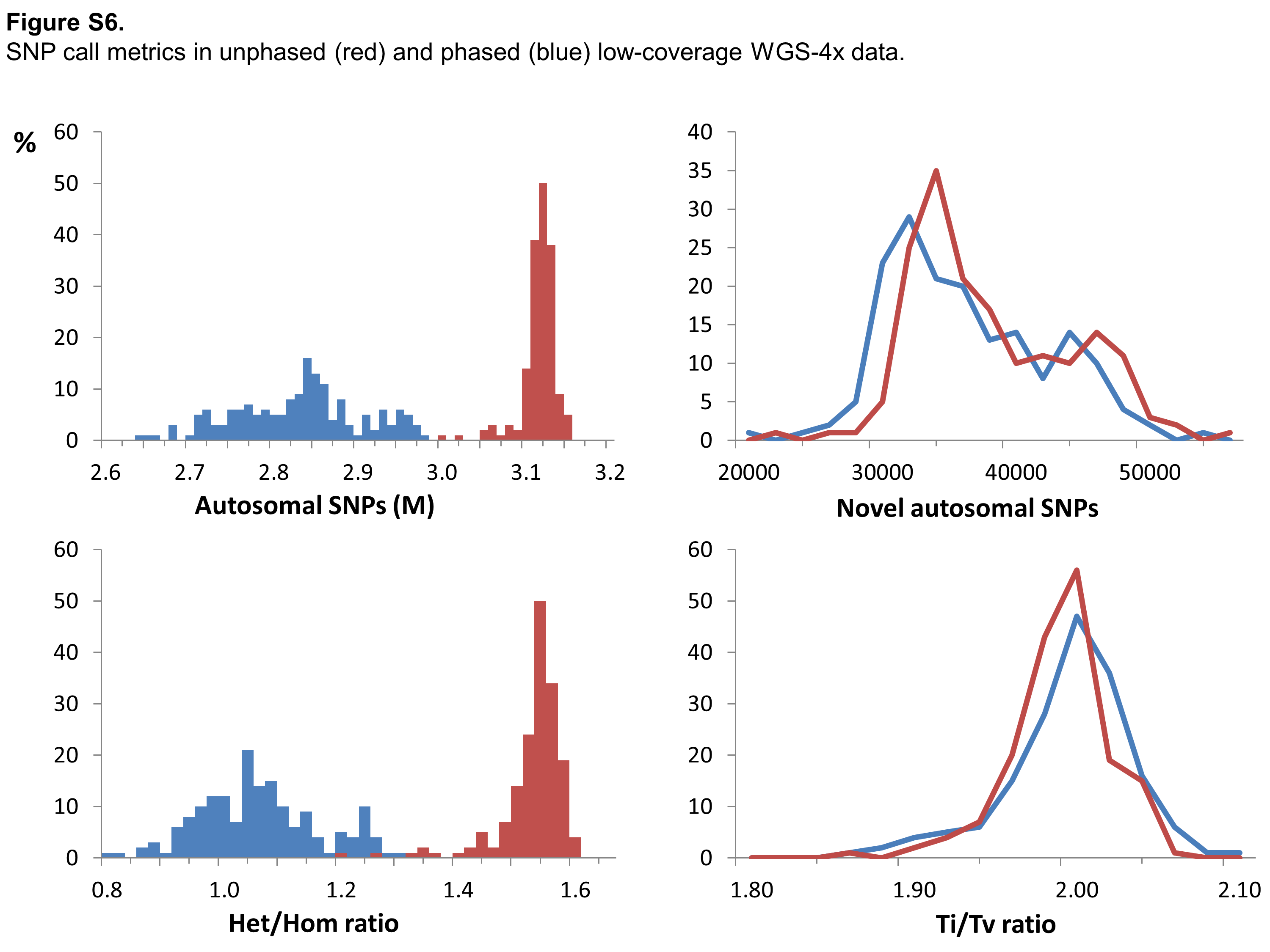

Supplement: Figure S6 — SNP call metrics in unphased (red) and phased (blue) low-coverage WGS-4x data. (TIF) [file pone.0102645.s006.tif]

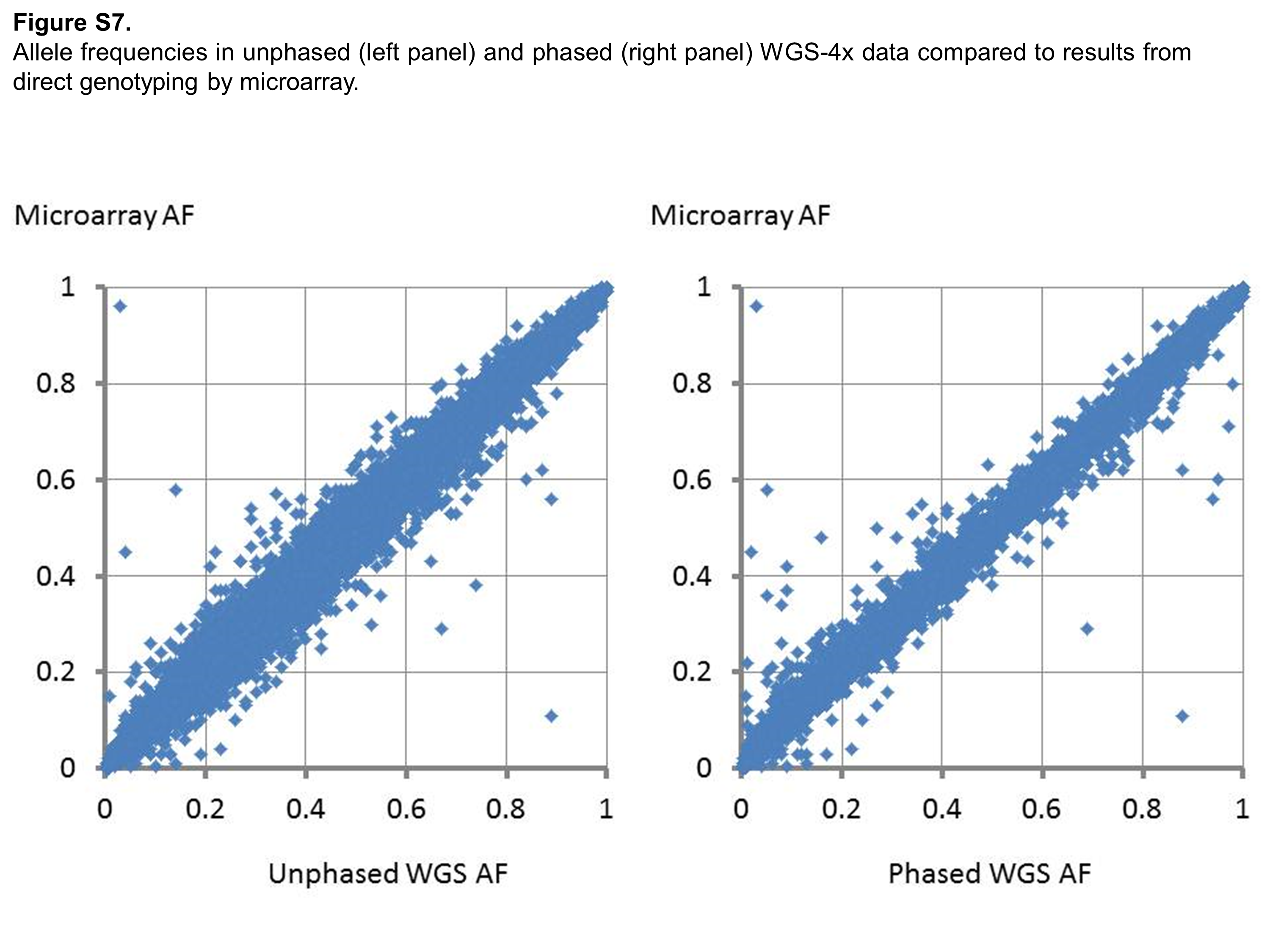

Supplement: Figure S7 — Allele frequencies in unphased (left panel) and phased (right panel) WGS-4x data compared to results from direct genotyping by microarray. (TIF) [file pone.0102645.s007.tif]

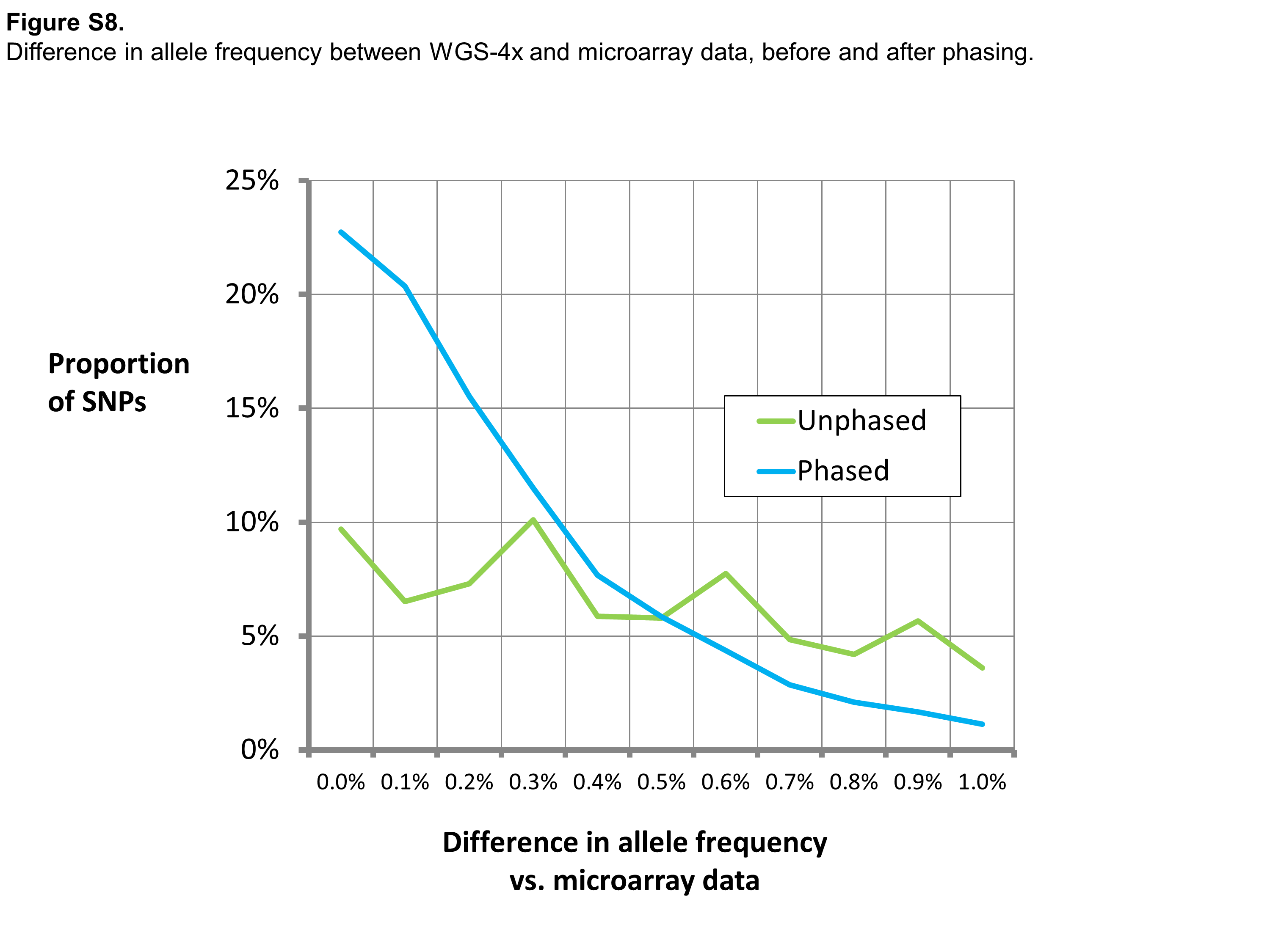

Supplement: Figure S8 — Difference in allele frequency between WGS-4x and microarray data, before and after phasing. (TIF) [file pone.0102645.s008.tif]

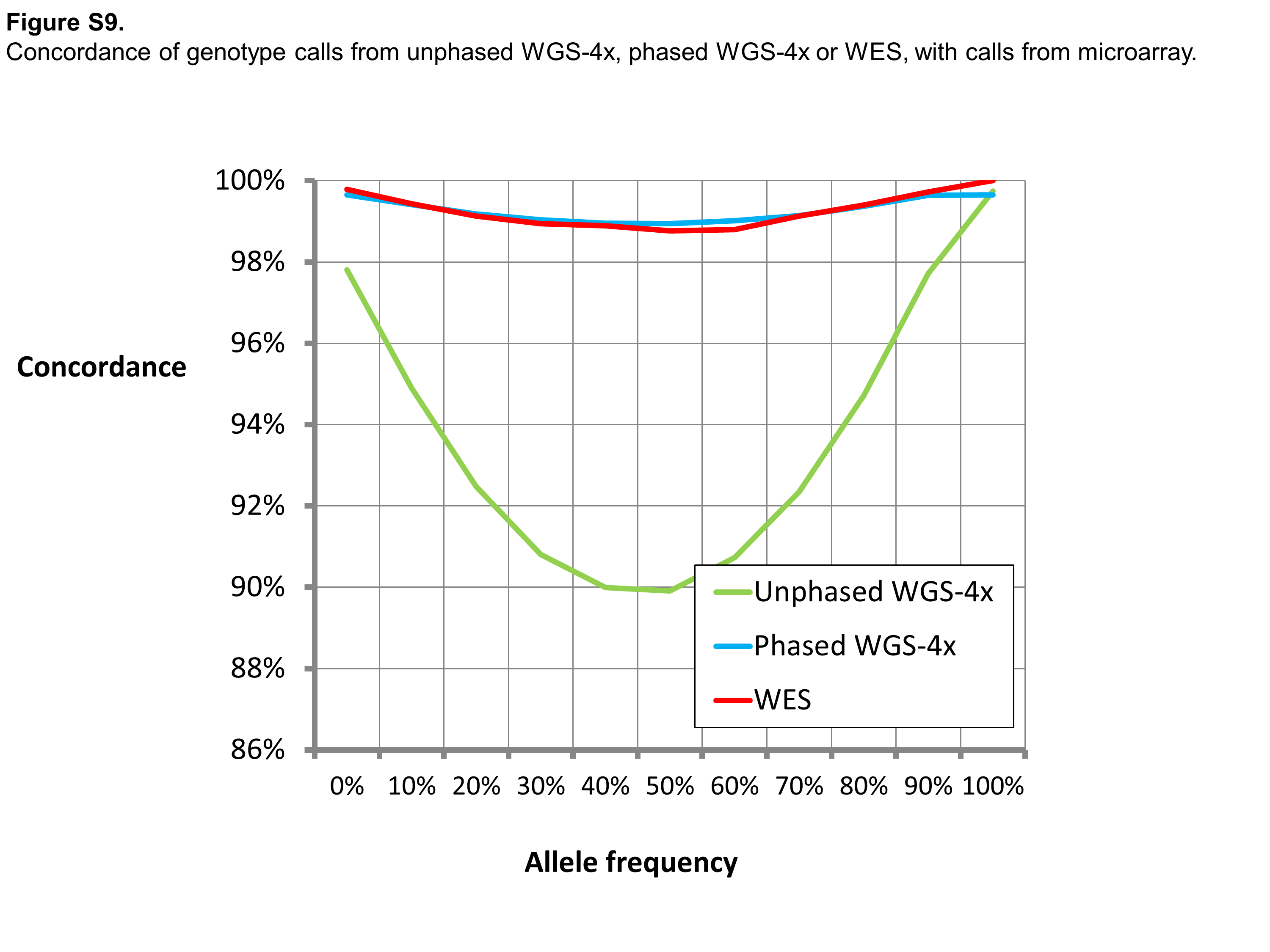

Supplement: Figure S9 — Concordance of genotype calls from unphased WGS-4x, phased WGS-4x or WES, with calls from microarray. (TIF) [file pone.0102645.s009.tif]

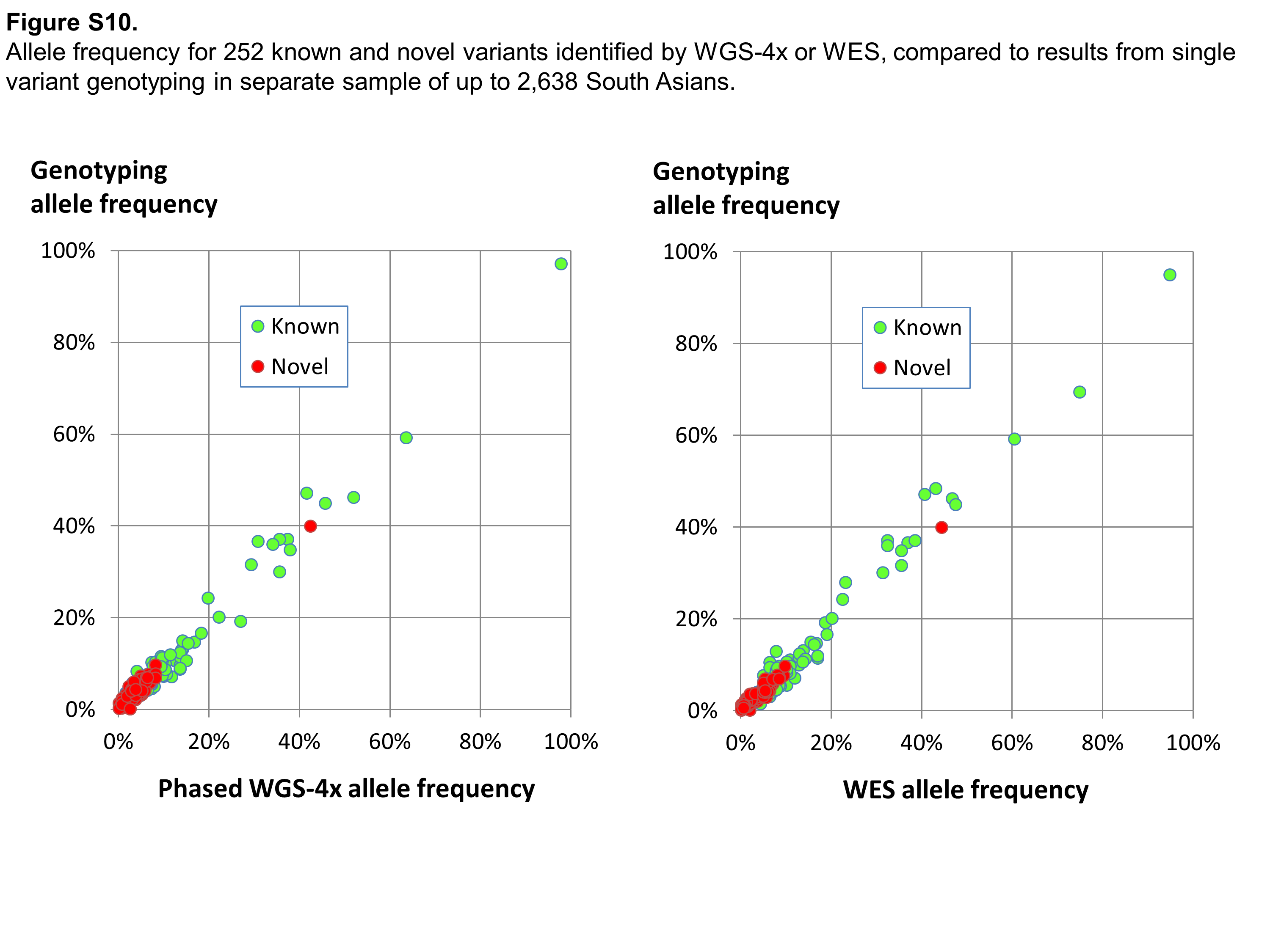

Supplement: Figure S10 — Allele frequency for 252 known and novel variants identified by WGS-4x or WES, compared to results from single variant genotyping in separate sample of up to 2,638 South Asians. (TIF) [file pone.0102645.s010.tif]

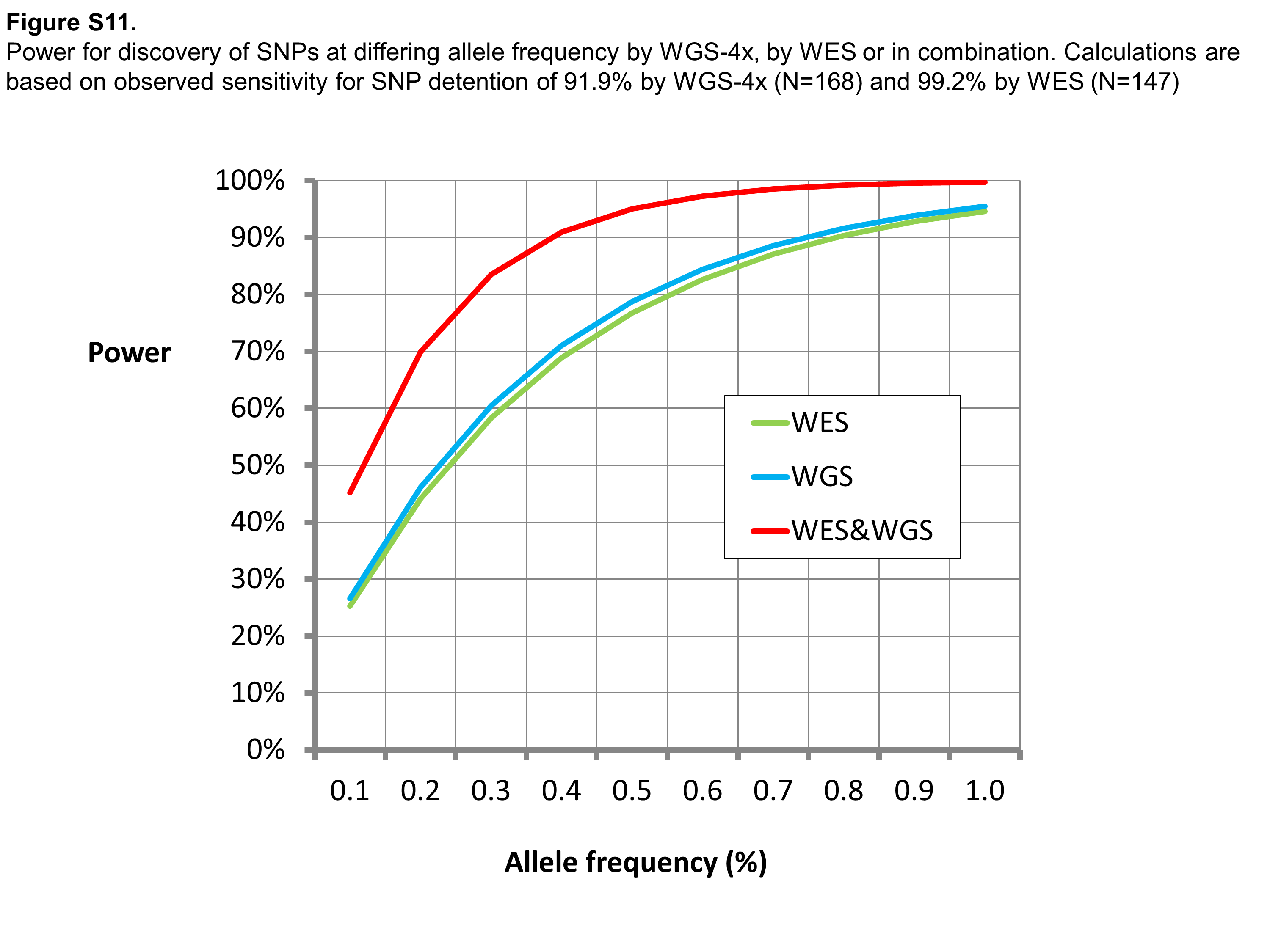

Supplement: Figure S11 — Power for discovery of SNPs at differing allele frequency by WGS-4x, by WES or in combination. Calculations are based on observed sensitivity for SNP detention of 91.9% by WGS-4x (N = 168) and 99.2% by WES (N = 147). (TIF) [file pone.0102645.s011.tif]

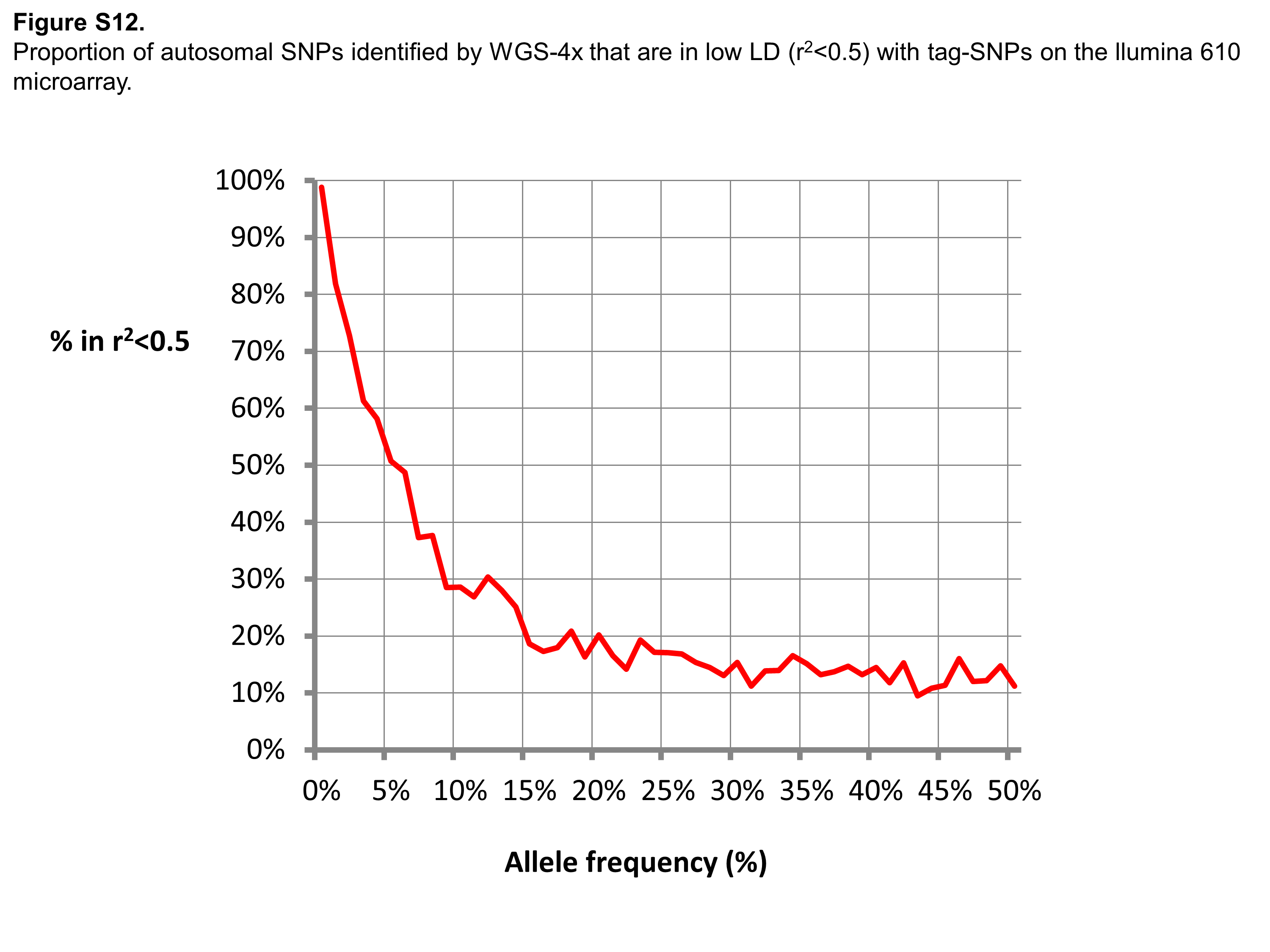

Supplement: Figure S12 — Proportion of autosomal SNPs identified by WGS-4x that are in low LD (r2<0.5) with tag-SNPs on the llumina 610 microarray. (TIF) [file pone.0102645.s012.tif]

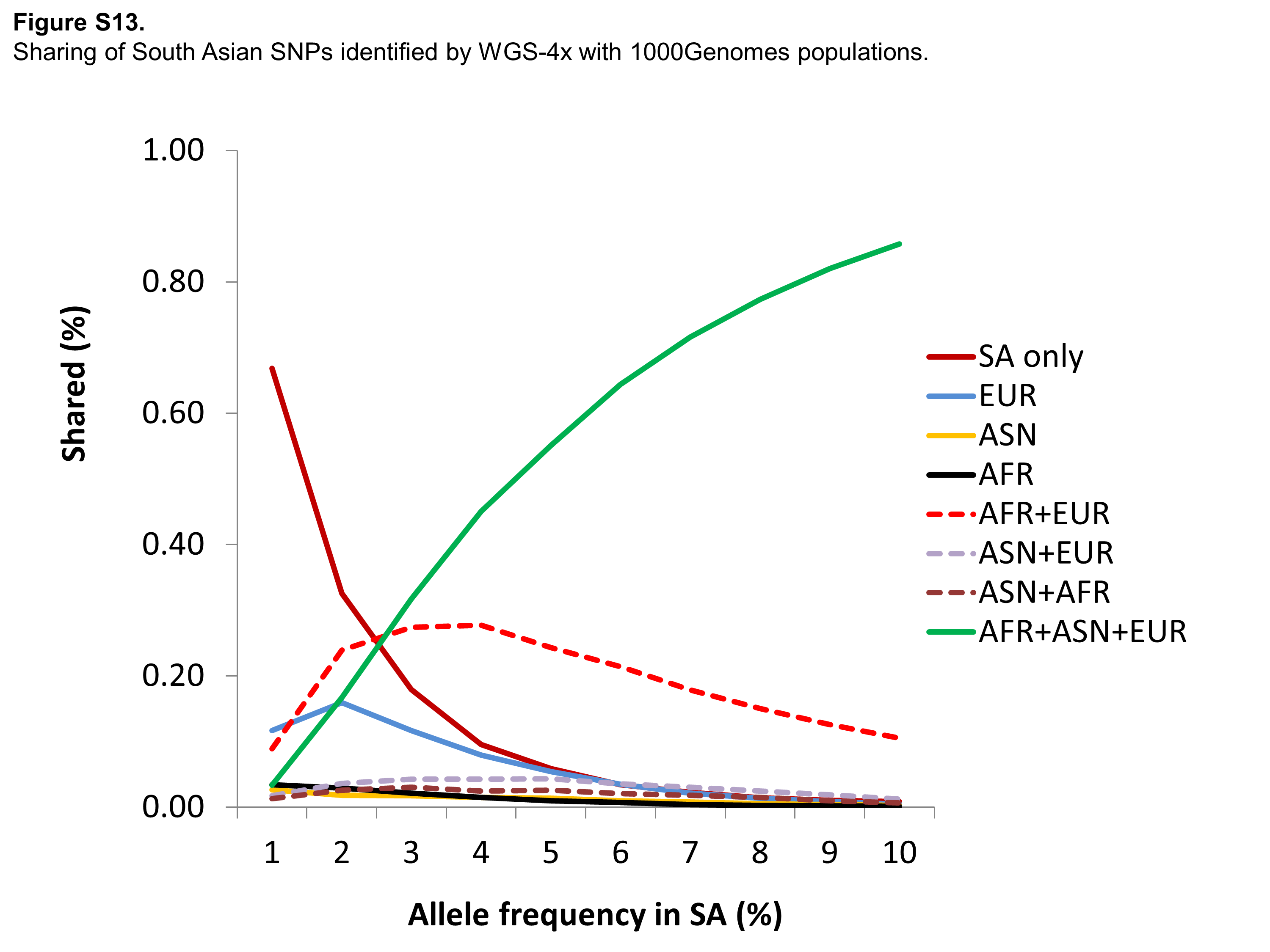

Supplement: Figure S13 — Sharing of South Asian SNPs identified by WGS-4x with 1000Genomes populations. (TIF) [file pone.0102645.s013.tif]

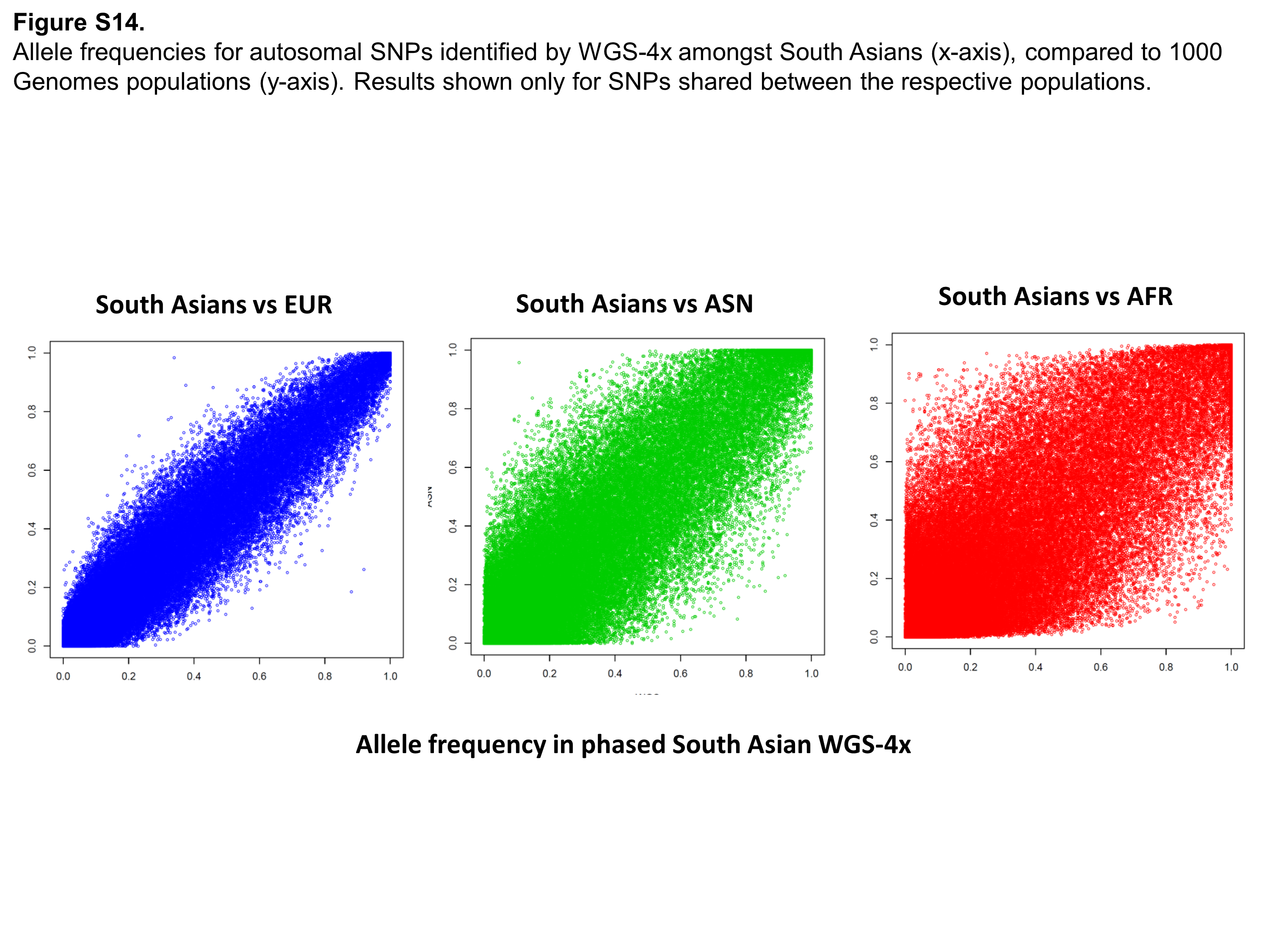

Supplement: Figure S14 — Allele frequencies for autosomal SNPs identified by WGS-4x amongst South Asians (x-axis), compared to 1000 Genomes populations (y-axis). Results shown only for SNPs shared between the respective populations. (TIF) [file pone.0102645.s014.tif]

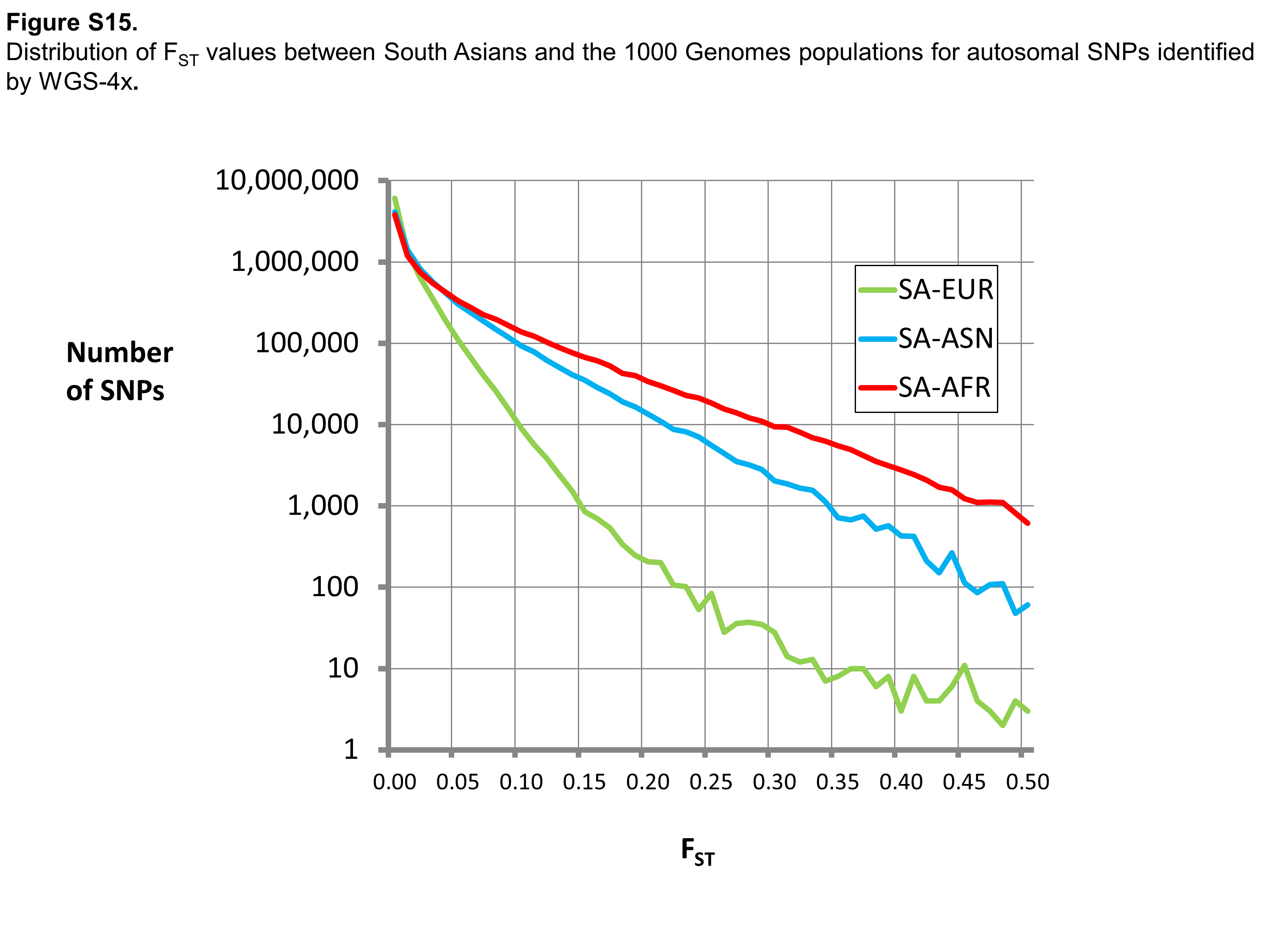

Supplement: Figure S15 — Distribution of FST values between South Asians and the 1000 Genomes populations for autosomal SNPs identified by WGS-4x. (TIF) [file pone.0102645.s015.tif]

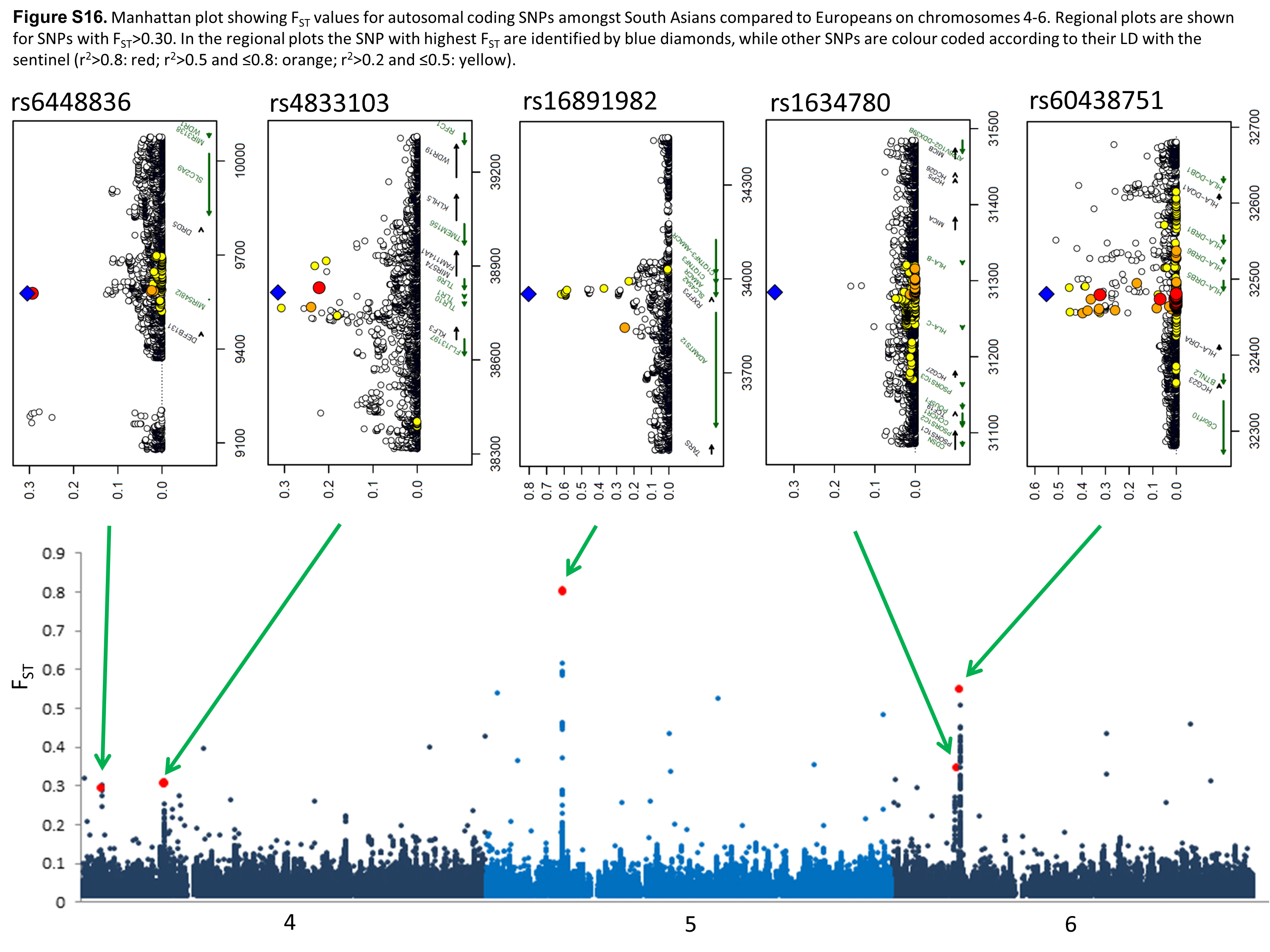

Supplement: Figure S16 — Manhattan plot showing FST values for autosomal coding SNPs amongst South Asians compared to Europeans on chromosomes 4–6. Regional plots are shown for SNPs with FST>0.30. In the regional plots the SNP with highest FST are identified by blue diamonds, while other SNPs are colour coded according to their LD with the sentinel (r2>0.8: red; r2>0.5 and ≤0.8: orange; r2>0.2 and ≤0.5: yellow). (TIF) [file pone.0102645.s016.tif]

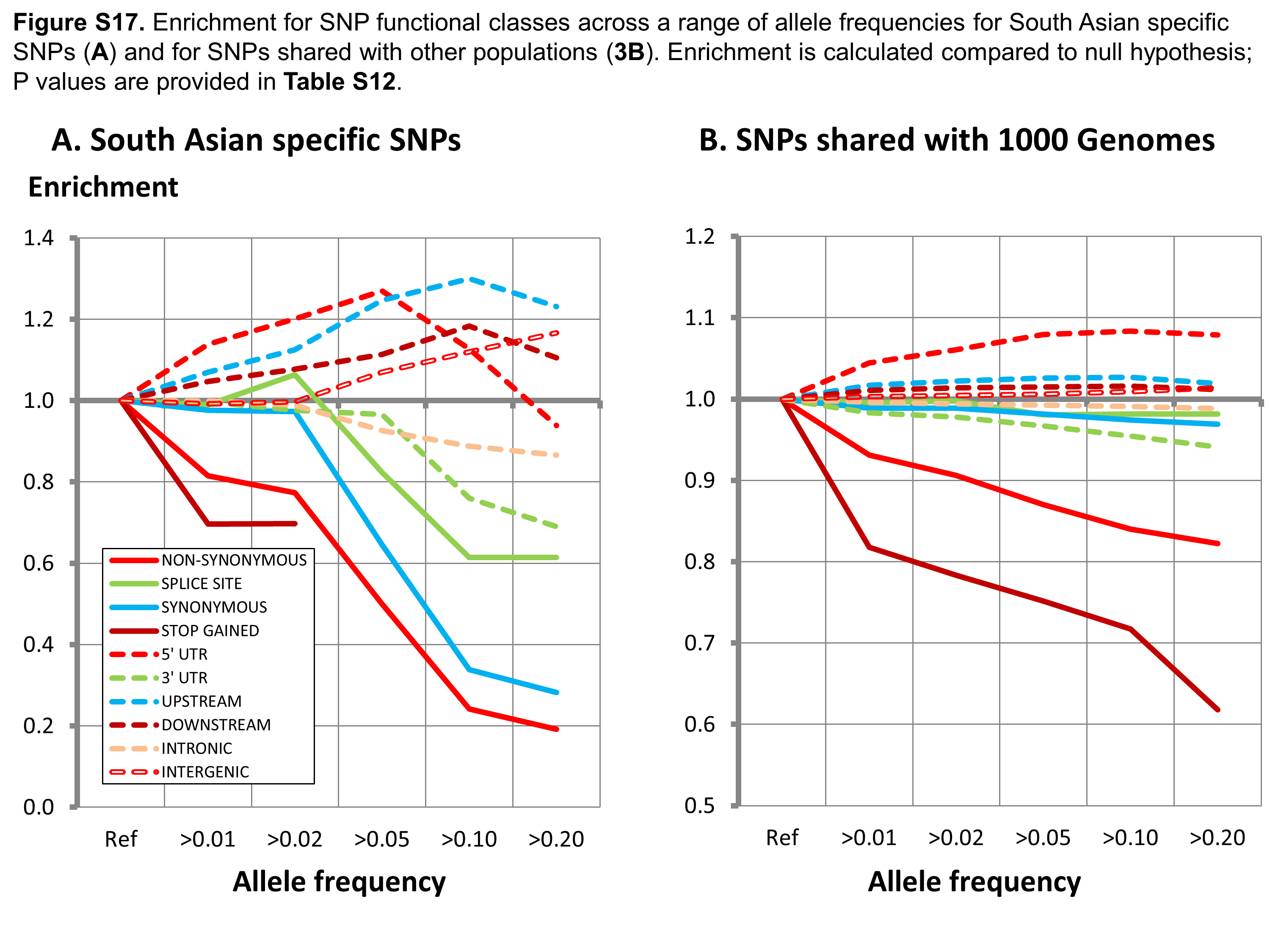

Supplement: Figure S17 — Enrichment for SNP functional classes across a range of allele frequencies for South Asian specific SNPs (A) and for SNPs shared with other populations (3B). Enrichment is calculated compared to null hypothesis; P values are provided in Table S12. (TIF) [file pone.0102645.s017.tif]

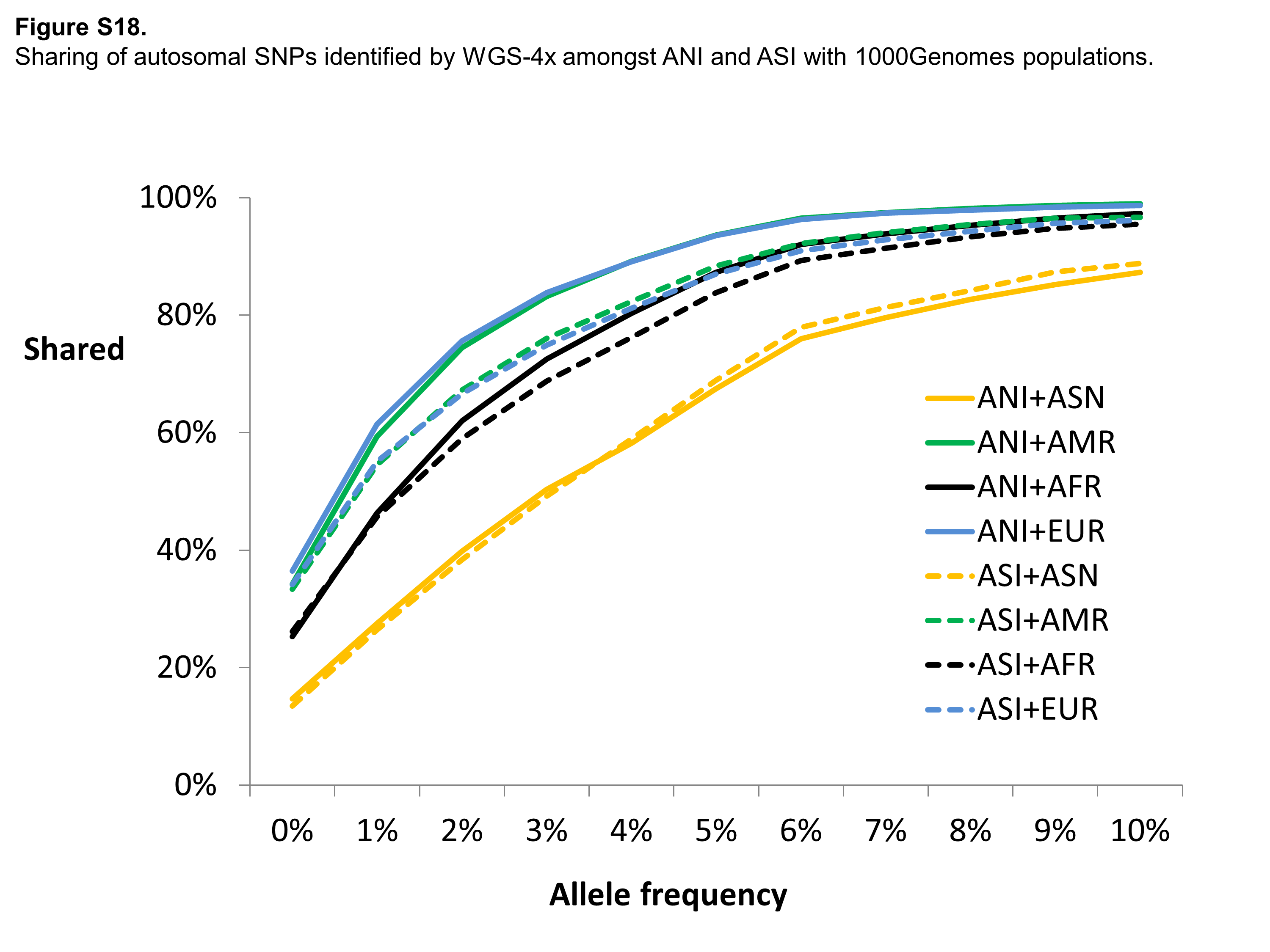

Supplement: Figure S18 — Sharing of autosomal SNPs identified by WGS-4x amongst ANI and ASI with 1000Genomes populations. (TIF) [file pone.0102645.s018.tif]

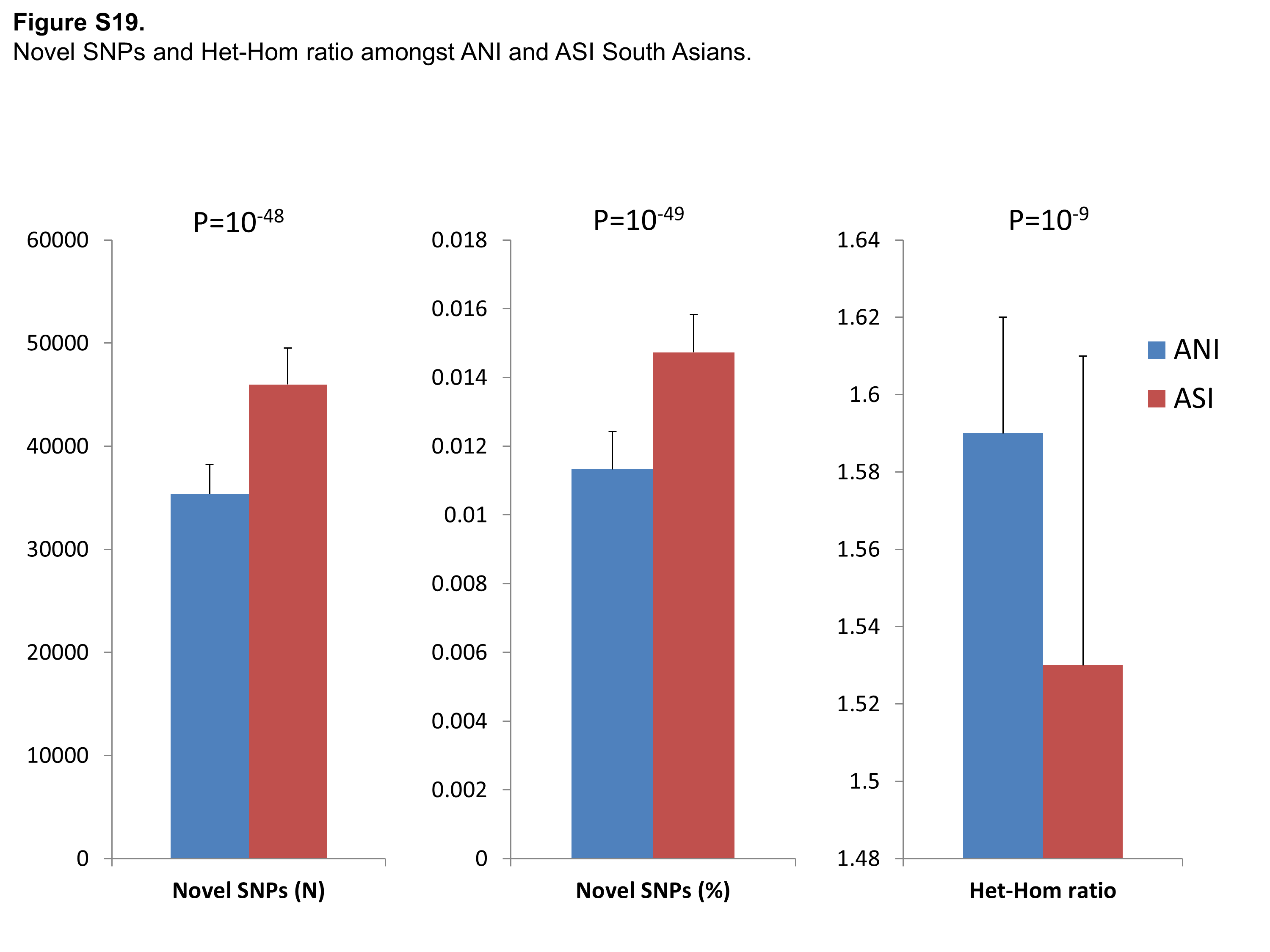

Supplement: Figure S19 — Novel SNPs and Het-Hom ratio amongst ANI and ASI South Asians. (TIF) [file pone.0102645.s019.tif]

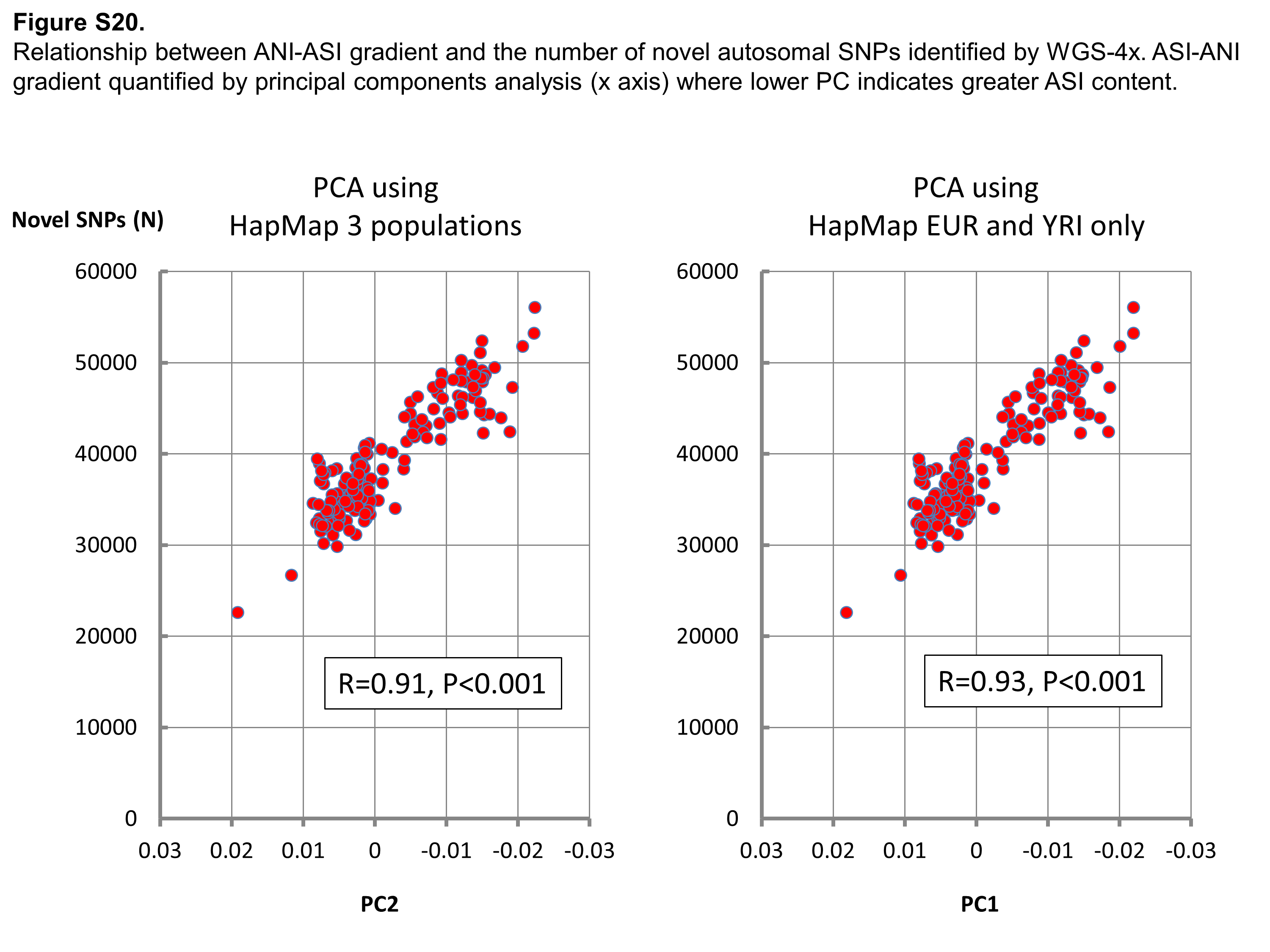

Supplement: Figure S20 — Relationship between ANI-ASI gradient and the number of novel autosomal SNPs identified by WGS-4x. ASI-ANI gradient quantified by principal components analysis (x axis) where lower PC indicates greater ASI content. (TIF) [file pone.0102645.s020.tif]
