# Supplementary material for: The South Asian Genome
Source: PLoS One. 2014 Aug 12;9(8):e102645. doi: 10.1371/journal.pone.0102645 (PMC4130493; doi:10.1371/journal.pone.0102645)
Supplement: File S1 — Table S1, Characteristics of participants. Table S2, Per sample sequencing metrics for WGS and WES. Results are mean (SD). Table S4, Results of indel validation by Sanger sequencing. Sanger sequencing of 35 indel (17 previously unreported) amongst 8 individuals predicted to carry the indels by WGS-28x. Sanger sequencing confirmed the presence of indels at all sites called by whole genome sequencing. For 33 of the 35 indels (94.3%) Sanger sequencing showed identical sequence to that predicted by WGS-28x. Sanger sequencing of the remaining two PCR amplicons confirmed presence of a complex indel within 20 nucleotides of the originally called indel; one fell within a repetitive region and the second was difficult to analyse. Both these indels were novel. Table S5, Functional class of SNPs identified by WGS-4x and WES. Table S6, Functional class of indels identified by WGS-4x and WES. Table S7, P values for enrichment of autosomal coding and intergenic SNPs across the range of FST between South Asians and the AFR, ASN or EUR populations. Table S8, P values for enrichment of functional classes amongst autosomal SNPs across the range of FST between South Asians and 1000 Genomes Project populations. Table S10, Pathway analysis (Ingenuity Pathway Analysis) of genes with potentially functional SNPs stratified between South Asians and Europeans (FST>0.10). Table S11, Enrichment for stratified SNPs at genetic loci known to be associated with respective phenotype in GWA studies. Observed: no of stratified SNPs (Fst>0.10) within 500 kb of the reported sentinel SNPs. Predicted: mean no of SNPs expected to fall within 500 kb of the sentinel SNPs under null hypothesis. Expectation based on permutation testing: 10,000 runs of SNP sets matched to the stratified SNPs based on allele frequency and gene proximity, but otherwise selected at random. Enrichment: observed/expected. P: exact probability of the observed based on the distribution of expected generated by permutation testing. Ta [file pone.0102645.s021.docx]

**Supplementary Online Material**

**The South Asian Genome**

John C Chambers, James Abbott, Weihua Zhang, Ernest Turro, William R Scott, Sian-Tsung Tan, Marie Loh, Benjamin Lehne, Paul O’Reilly, Kyle J Gaulton, Richard D Pearson, Xinzhong Li, Anita Lavery, Jana Vandrovcova, Mark N Wass, Kathryn Miller, Joban Sehmi, Laticia Oozageer, Ishminder K Kooner, Abtehale Al-Hussaini, Uzma Afzal, Rebecca Mills, Jagvir Grewal, Vasileios Panoulas, Alexandra M Lewin, Korrinne Northwood, Gurpreet S Wander, Frank Geoghegan, Yingrui Li, Jun Wang, Timothy J Aitman, Mark I McCarthy, James Scott, Sarah Butcher, Paul Elliott and Jaspal S Kooner.

**Table S1**. Characteristics of participants.

|  | **WGS-4x** | **WGS-28x** | **WES** |
| --- | --- | --- | --- |
|  |  |  |  |
| *N* | 168 | 8 | 147 |
| Male (%) | 89.3 | 100 | 89.8 |
|  |  |  |  |
| *Language* |  |  |  |
| Gujarati | 10 | 0 | 7 |
| Hindi | 16 | 2 | 7 |
| Konkani | 9 | 0 | 0 |
| Malayalam | 15 | 0 | 3 |
| Marathi | 8 | 0 | 0 |
| Punjabi | 91 | 1 | 64 |
| Sinhalese | 0 | 1 | 4 |
| Tamil | 7 | 1 | 10 |
| Urdu | 10 | 1 | 33 |
| Other | 2 | 1 | 17 |
|  |  |  |  |
| *Religion* |  |  |  |
| Christian | 18 | 0 | 9 |
| Hindu | 80 | 4 | 34 |
| Moslem | 16 | 2 | 47 |
| Sikh | 51 | 1 | 52 |
| Other | 3 | 1 | 5 |
|  |  |  |  |
| Age (years) | 57.2 (10.8) | 57.8 (9.6) | 44.9 (5.1) |
| UK years | 27.2 (14.4) | 26.0 (17.0) | 21.6 (11.4) |
| Type-2 diabetes (%) | 28 | 25 | 16 |
| Coronary heart disease (%) | 10 | 13 | 5 |
| Hypertension (%) | 42 | 38 | 35 |
| Systolic blood pressure (mmHg) | 137.8 (19.9) | 131.5 (18.8) | 129.8 (19.6) |
| Diastolic blood pressure (mmHg) | 82.4 (10.6) | 81.3 (12.3) | 80.8 (11.9) |
| Body mass index (kg/m^2^) | 26.7 (4.2) | 26.2 (4.6) | 25.5 (4.2) |
| Waist-hip ratio | 0.96 (0.07) | 0.96 (0.05) | 0.95 (0.06) |
| Cholesterol (mmol/l) | 5.17 (1.19) | 4.89 (1.06) | 4.94 (1.20) |
| HDL cholesterol (mmol/l) | 1.21 (0.25) | 1.23 (0.22) | 1.17 (0.32) |
| Triglycerides (mmol/l) | 1.82 (1.13) | 1.32 (0.62) | 2.09 (1.41) |
|  |  |  |  |

**Table S2**. Per sample sequencing metrics for WGS and WES. Results are mean (SD).

|  | **WGS-28x** | **WGS-4x** | **WES** |
| --- | --- | --- | --- |
|  |  |  |  |
| Total reads (millions) | 1471 (143) | 172 (19) | 42.3 (20.1) |
| Duplicates reads (millions) | 423 (251) | 12.1 (3.5) | 13.0 (12.2) |
| Duplicates (%) | 31.7 (16.4) | 7.2 (2.4) | 26.5 (15.5) |
| Mapped reads (millions) | 1305 (106) | 163 (19) | 40.3 (18.9) |
| Mapped (%) | 88.9 (3.6) | 94.5 (0.9) | 95.7 (1.7) |
| Mean coverage of genome / target | 28.4 (6.2) | 4.3 (0.6) | 20.6 (7.6) |
| % Genome/target mapped at >1x | 92.3 (0.1) | 89.6 (0.8) | 94.5 (1.5) |
|  |  |  |  |
| Autosomal SNP TiTv ratio | 2.12 (0.01) | 2.13 (0.01) | 2.65 (0.06) |
| Autosomal SNP Het/Hom ratio | 1.55 (0.04) | 1.57 (0.06) | 1.39 (0.10) |
|  |  |  |  |

**Table S3**. Replication results for 252 SNPs genotyped by single variant tests amongst up to 2,638 South Asians. Dataset: sequence data SNP was selected from. AF: allele frequency.

[Excel file]

**Table S4**. Results of indel validation by Sanger sequencing. Sanger sequencing of 35 indel (17 previously unreported) amongst 8 individuals predicted to carry the indels by WGS-28x. Sanger sequencing confirmed the presence of indels at all sites called by whole genome sequencing . For 33 of the 35 indels (94.3%) Sanger sequencing showed identical sequence to that predicted by WGS-28x. Sanger sequencing of the remaining two PCR amplicons confirmed presence of a complex indel within 20 nucleotides of the originally called indel; one fell within a repetitive region and the second was difficult to analyse. Both these indels were novel.

| **Chr** | **Position** | **Reference** | **Alternative*** | **Confirmed by Sanger Sequencing** | **WGS Allele Frequency** | **Sanger Allele Frequency** |
| --- | --- | --- | --- | --- | --- | --- |
| 1 | 34421405 | CAAAAT | C | Yes | 0.563 | 0.563 |
| 1 | 39071780 | G | GA | Yes | 0.063 | 0.063 |
| 1 | 57227460 | CCTCT | C | Yes | 0.438 | 0.438 |
| 1 | 63570931 | AATGGGATTCTAGGAT | A | Yes | 0.063 | 0.063 |
| 1 | 173237052 | A | AAGGAAC | Yes | 0.125 | 0.125 |
| 1 | 229302642 | GA | G | Yes | 0.063 | 0.063 |
| 2 | 70513648 | C | CT | Yes | 0.063 | 0.063 |
| 2 | 107231589 | TAGG | T | Yes | 0.063 | 0.063 |
| 2 | 239331251 | ACC | A | Yes | 0.063 | 0.063 |
| 3 | 10434045 | TGCCCTGTCCTCTCCCAG | T | Yes | 0.063 | 0.063 |
| 3 | 24863547 | T | TA | Yes | 0.063 | 0.063 |
| 3 | 51385959 | AAAGT | A | Yes | 0.063 | 0.063 |
| 3 | 109344848 | G | GA | Yes | 0.063 | 0.063 |
| 3 | 193964511 | TCATGAAAGTCTCCCTCCAC | T | Yes | 0.063 | 0.063 |
| 4 | 6388911 | TA | T | Yes | 0.063 | 0.063 |
| 5 | 71035744 | AAG | A | Yes | 0.125 | 0.125 |
| 5 | 163198560 | GT | G | Yes | 0.125 | 0.125 |
| 5 | 163198560 | G | GAAAA | Yes | 0.813 | 0.813 |
| 6 | 85759289 | C | CT | Complex deletion identified in the alternative allele at this locus | 0.063 | 0.063 |
| 7 | 155599630 | CGCCCCCACGTTGCCCTCCACTTCTA | C | Yes | 0.063 | 0.063 |
| 10 | 90335467 | GATAA | G | Yes | 0.063 | 0.063 |
| 11 | 10725946 | TA | T | Yes | 0.063 | 0.063 |
| 12 | 18570680 | TTAAAAAAA | T | Yes | 0.063 | 0.063 |
| 12 | 20851211 | CTTCT | C | Yes | 0.063 | 0.063 |
| 13 | 24690876 | TC | T | Yes | 0.063 | 0.063 |
| 13 | 63883368 | A | AT | Yes | 0.375 | 0.375 |
| 14 | 40301837 | T | TA | Yes | 0.063 | 0.063 |
| 14 | 40657690 | T | TA | Yes | 0.125 | 0.125 |
| 17 | 20200129 | CTCTG | C | Yes | 0.063 | 0.063 |
| 17 | 26001819 | CTTTTAGTATCTCTAATGGTCT | C | 21 base pair deletion identified in the alternate allele at 17: 26001839 | 0.063 | 0.063 |
| 18 | 21375698 | T | TA | Yes | 0.063 | 0.063 |
| 18 | 43724710 | T | TA | Yes | 0.063 | 0.063 |
| 18 | 76987962 | CATAT | C | Yes | 0.063 | 0.063 |
| 19 | 8221572 | A | AT | Yes | 0.75 | 0.75 |
| 22 | 40405215 | TC | T | Yes | 0.063 | 0.063 |

**Table S5**. Functional class of SNPs identified by WGS-4x and WES.

|  | **Autosomal** | | | |  | **Sex chromosome** | | | |
| --- | --- | --- | --- | --- | --- | --- | --- | --- | --- |
| **Functional Class** | **WGS-4x** | **WES** | **WGS-4x**  **& WES** | **Novel** |  | **WGS** | **WES** | **WGS-4x & WES** | **Novel** |
|  |  |  |  |  |  |  |  |  |  |
| 3PRIME_UTR | 104,409 | 80,430 | 138,773 | 54,496 |  | 2,253 | 1,929 | 3,167 | 1,458 |
| 5PRIME_UTR | 23,294 | 7,488 | 26,629 | 7,030 |  | 425 | 311 | 575 | 218 |
| DOWNSTREAM | 420,835 | 2,551 | 421,843 | 99,963 |  | 13,964 | 43 | 13,982 | 4,216 |
| INTERGENIC | 4,577,834 | 545 | 4,578,040 | 1,129,682 |  | 219,040 | 38 | 219,055 | 67,317 |
| INTRONIC | 5,772,141 | 6,511 | 5,774,749 | 1,477,868 |  | 113,541 | 124 | 113,609 | 37,312 |
| NON_SYNONYMOUS_CODING | 45,201 | 48,104 | 70,746 | 30,914 |  | 813 | 1,074 | 1,469 | 753 |
| PARTIAL_CODON | 6 | 2 | 6 | 2 |  |  |  |  |  |
| SPLICE_SITE | 12,657 | 1,587 | 13,332 | 3,623 |  | 218 | 43 | 240 | 80 |
| STOP_GAINED | 746 | 742 | 1,270 | 695 |  | 17 | 21 | 27 | 15 |
| STOP_LOST | 135 | 85 | 175 | 62 |  | 4 | 4 | 5 | 2 |
| SYNONYMOUS_CODING | 35,812 | 36,842 | 51,190 | 16,617 |  | 718 | 876 | 1,137 | 447 |
| UPSTREAM | 492,467 | 933 | 492,859 | 11,270,1 |  | 15,625 | 28 | 15,642 | 4,779 |
| WITHIN_MATURE_miRNA | 106 | 14 | 111 | 31 |  | 5 | 1 | 6 | 3 |
| WITHIN_NON_CODING_GENE | 53,246 | 4,105 | 54,893 | 13,177 |  | 1,723 | 168 | 1,805 | 597 |
|  |  |  |  |  |  |  |  |  |  |
| **Total** | **11,538,889** | **189,939** | **11,624,616** | **2,946,861** |  | **368,346** | **4,660** | **370,719** | **117,197** |
|  |  |  |  |  |  |  |  |  |  |
|  |  |  |  |  |  |  |  |  |  |

**Table S6**. Functional class of indels identified by WGS-4x and WES.

|  | **Autosomal** | | |  | | | **Sex chromosomes** | | |
| --- | --- | --- | --- | --- | --- | --- | --- | --- | --- |
| **CLASS** | **WGS-4x** | **WES** | **WGS-4x**  **& WES** | **Novel** |  | **WGS-4x** | **WES** | **WGS-4x**  **& WES** | **Novel** |
|  |  |  |  |  |  |  |  |  |  |
| Downstream | 15,845 | 478 | 16,196 | 4,015 |  | 585 | 7 | 588 | 251 |
| Exonic | 1,734 | 2,185 | 3,908 | 1,441 |  | 34 | 91 | 125 | 67 |
| Exonic & splicing | 82 | 63 | 144 | 48 |  | 2 | 3 | 5 | 4 |
| Frameshift deletion | 304 | 888 | 1,052 | 721 |  | 6 | 16 | 18 | 13 |
| Frameshift insertion | 283 | 429 | 594 | 331 |  | 13 | 5 | 15 | 6 |
| Intergenic | 608,211 | 581 | 604,775 | 135,553 |  | 27,202 | 27 | 27,080 | 10,885 |
| Intronic | 530,521 | 494 | 526,733 | 119,717 |  | 11,822 | 11 | 11,785 | 4,476 |
| ncRNA_exonic | 6,294 | 1,386 | 7,628 | 2,185 |  | 142 | 20 | 160 | 70 |
| ncRNA_intronic | 145,114 | 265 | 144,414 | 32,614 |  | 2,042 | 2 | 2,032 | 715 |
| ncRNA_splicing | 74 | 10 | 84 | 19 |  | 2 |  | 2 |  |
| ncRNA_UTR3 | 895 | 1,206 | 2,096 | 807 |  | 12 | 12 | 24 | 11 |
| ncRNA_UTR5 | 153 | 81 | 233 | 53 |  |  | 2 | 2 | 1 |
| ncRNA_UTR5;ncRNA_UTR3 | 3 | 1 | 4 | 2 |  |  |  |  |  |
| Nonframeshift deletion | 271 | 866 | 931 | 562 |  | 5 | 29 | 30 | 22 |
| Nonframeshift insertion | 170 | 313 | 356 | 166 |  | 5 | 11 | 11 | 6 |
| Splicing | 134 | 58 | 188 | 55 |  | 3 | 4 | 7 | 1 |
| Stopgain SNV | 10 | 28 | 33 | 21 |  |  |  |  |  |
| Stoploss SNV | 6 | 7 | 9 | 4 |  |  | 1 | 1 | 1 |
| Unknown | 38 | 28 | 54 | 19 |  |  |  |  |  |
| Upstream | 13,251 | 72 | 13,206 | 3,161 |  | 425 | 7 | 429 | 215 |
| Upstream & downstream | 752 | 30 | 771 | 203 |  | 16 |  | 16 | 4 |
| UTR3 | 11,250 | 15,081 | 26,230 | 10,041 |  | 254 | 456 | 709 | 409 |
| UTR5 | 1,866 | 1,185 | 3,031 | 991 |  | 40 | 42 | 82 | 44 |
| UTR5 & UTR3 | 22 | 15 | 36 | 9 |  |  |  |  |  |
|  |  |  |  |  |  |  |  |  |  |
| **Total** | **1,337,283** | **25,750** | **1,352,706** | **312,738** |  | **42,610** | **746** | **43,121** | **17,201** |
|  |  |  |  |  |  |  |  |  |  |

**Table S7**. P values for enrichment of autosomal coding and intergenic SNPs across the range of F_ST_ between South Asians and the AFR, ASN or EUR populations.

| **F_ST_ >** | **0.05** | **0.10** | **0.15** | **0.20** | **0.25** | **0.30** | **0.35** | **0.40** |
| --- | --- | --- | --- | --- | --- | --- | --- | --- |
| *Coding SNPs* | |  |  |  |  |  |  |  |
| AFR | 1.4E-01 | 7.5E-01 | 1.2E-05 | 6.2E-04 | 1.3E-06 | 7.6E-05 | 1.5E-03 | 1.4E-03 |
| ASN | 9.8E-01 | 4.1E-03 | 7.8E-03 | 2.1E-04 | 1.8E-06 | 7.4E-04 | 2.9E-03 | 1.1E-04 |
| EUR | 2.4E-08 | 1.4E-13 | 1.8E-15 | 4.7E-13 | 5.6E-04 |  |  |  |
| *Intergenic SNPs* | |  |  |  |  |  |  |  |
| AFR | 1.2E-04 | 1.2E-34 | 4.0E-51 | 6.9E-76 | 2.1E-97 | 7.9E-81 | 1.2E-77 | 1.2E-81 |
| ASN | 4.2E-63 | 3.6E-79 | 1.2E-48 | 3.8E-82 | 1.0E-55 | 1.5E-63 | 6.6E-22 | 7.1E-08 |
| EUR | 1.9E-284 | 5.4E-123 | 2.4E-43 | 3.0E-15 | 4.8E-04 |  |  |  |

**Table S8**. P values for enrichment of functional classes amongst autosomal SNPs across the range of F_ST_ between South Asians and 1000 Genomes Project populations.

| **F_ST_ >** | **0.05** | **0.10** | **0.15** | **0.20** | **0.25** |
| --- | --- | --- | --- | --- | --- |
|  |  |  |  |  |  |
| **South Asians vs EUR** |  |  |  |  |  |
| NON_SYNONYMOUS_CODING | 1.1E-01 | 9.4E-07 | 3.4E-03 | 1.1E-03 | 1.3E-05 |
| SPLICE_SITE | 1.7E-01 | 1.9E-02 | 7.4E-08 | 1.4E-04 |  |
| SYNONYMOUS_CODING | 2.8E-07 | 1.2E-04 | 6.8E-06 | 2.0E-05 |  |
| STOP_GAINED | 9.9E-01 | 7.6E-01 |  |  |  |
| 5PRIME_UTR | 2.1E-17 | 2.6E-07 | 1.2E-03 | 1.9E-04 | 7.0E-05 |
| 3PRIME_UTR | 3.2E-05 | 4.4E-09 | 3.3E-01 | 8.5E-01 |  |
| UPSTREAM | 1.1E-26 | 3.5E-12 | 7.2E-05 | 7.2E-02 | 4.0E-01 |
| DOWNSTREAM | 2.5E-02 | 1.2E-01 | 7.3E-01 | 6.8E-02 | 9.9E-01 |
| INTRONIC | 4.7E-154 | 1.9E-67 | 2.3E-17 | 9.4E-04 | 3.0E-01 |
| INTERGENIC | 1.9E-284 | 5.4E-123 | 2.4E-43 | 3.0E-15 | 4.8E-04 |
|  |  |  |  |  |  |
| **South Asians vs ASN** |  |  |  |  |  |
| NON_SYNONYMOUS_CODING | 6.0E-03 | 7.9E-01 | 9.1E-01 | 2.8E-01 | 2.5E-02 |
| SPLICE_SITE | 1.7E-02 | 3.4E-03 | 9.2E-02 | 7.6E-03 | 4.2E-02 |
| SYNONYMOUS_CODING | 7.6E-01 | 2.9E-04 | 3.1E-04 | 5.7E-02 | 4.7E-03 |
| STOP_GAINED | 1.0E+00 | 9.9E-01 |  |  |  |
| 5PRIME_UTR | 2.3E-03 | 2.7E-03 | 2.8E-02 | 1.1E-02 | 1.7E-02 |
| 3PRIME_UTR | 1.0E+00 | 6.6E-02 | 1.4E-04 | 2.4E-04 | 8.5E-04 |
| UPSTREAM | 9.7E-01 | 7.1E-01 | 9.8E-01 | 6.2E-01 | 1.9E-01 |
| DOWNSTREAM | 9.0E-01 | 6.5E-01 | 2.1E-01 | 6.9E-01 | 2.5E-01 |
| INTRONIC | 2.2E-56 | 4.7E-65 | 1.5E-30 | 1.1E-56 | 2.6E-30 |
| INTERGENIC | 4.2E-63 | 3.6E-79 | 1.2E-48 | 3.8E-82 | 1.0E-55 |
|  |  |  |  |  |  |
| **South Asians vs AFR** |  |  |  |  |  |
| NON_SYNONYMOUS_CODING | 8.2E-07 | 2.0E-01 | 7.2E-01 | 4.9E-01 | 1.6E-01 |
| SPLICE_SITE | 6.7E-01 | 1.3E-01 | 1.5E-02 | 9.1E-03 | 6.1E-04 |
| SYNONYMOUS_CODING | 5.1E-01 | 8.9E-02 | 3.5E-05 | 2.6E-02 | 8.4E-04 |
| STOP_GAINED | 4.1E-01 | 4.5E-01 | 7.3E-01 | 7.9E-01 |  |
| 5PRIME_UTR | 2.1E-05 | 7.7E-04 | 6.3E-03 | 5.7E-02 | 7.2E-02 |
| 3PRIME_UTR | 4.0E-02 | 9.6E-01 | 5.9E-01 | 1.5E-02 | 4.7E-02 |
| UPSTREAM | 6.6E-07 | 5.5E-06 | 3.1E-01 | 4.6E-01 | 3.5E-01 |
| DOWNSTREAM | 2.0E-04 | 1.3E-04 | 1.9E-02 | 4.8E-03 | 1.1E-03 |
| INTRONIC | 8.9E-01 | 7.0E-13 | 7.2E-29 | 2.7E-46 | 8.6E-59 |
| INTERGENIC | 1.2E-04 | 1.2E-34 | 4.0E-51 | 6.9E-76 | 2.1E-97 |
|  |  |  |  |  |  |

**Table S9**. Coding, splice and UTR SNPs showing greatest stratification between South Asians and Europeans. AF: allele frequency, SA: South Asians, AFR; Africans, ASN: East Asians; EUR: Europeans.

[Excel file]

**Table S10**. Pathway analysis (Ingenuity Pathway Analysis) of genes with potentially functional SNPs stratified between South Asians and Europeans (F_ST_>0.10).

| **Annotation** | **P** | **N** | **Genes** |
| --- | --- | --- | --- |
|  |  |  |  |
| *Cellular Development* |  |  |  |
| Proliferation of immune cells | 1.8E-03 | 26 | *BSG, CD33, CD3G, CD6, DLG1, EOMES, EPHB1, ETS2, FN1 (includes EG:100005469), GAB2, HLA-DQA1, ICOSLG, IFNGR1, IL5RA, ITGAL, LAT, MLLT3, PAWR, PBX1, PIK3CD, POU2AF1, SH2B3, TNFRSF4, TYR, TYRP1, VDR* |
| Proliferation of blood cells | 1.9E-03 | 27 | *BSG, CD33, CD3G, CD6, DLG1, EOMES, EPHB1, ETS2, FN1 (includes EG:100005469), GAB2, HLA-DQA1, ICOSLG, IFNGR1, IL5RA, ITGAL, LAT, MECOM, MLLT3, PAWR, PBX1, PIK3CD, POU2AF1, SH2B3, TNFRSF4, TYR, TYRP1, VDR* |
| Proliferation of lymphocytes | 2.9E-03 | 24 | *BSG, CD33, CD3G, CD6, DLG1, EOMES, EPHB1, ETS2, FN1 (includes EG:100005469), GAB2, HLA-DQA1, ICOSLG, IFNGR1, IL5RA, ITGAL, LAT, PAWR, PIK3CD, POU2AF1, SH2B3, TNFRSF4, TYR, TYRP1, VDR* |
| Differentiation of endocrine cells | 3.0E-03 | 3 | *NEUROD1, PBX1, PCSK2* |
| Development of lymphocytes | 5.6E-03 | 18 | *BSG, CD3G, CD6, CSK, EOMES, ETS2, GAS6, HLA-DQA1, ICOSLG, IFNGR1, IL5RA, ITGA4, ITGAL, LAT, PGF, PIK3CD, POU2AF1, TNFRSF4* |
| Differentiation of melanocytes | 8.2E-03 | 3 | *HPS4 (includes EG:192232), OCA2 (includes EG:18431), TYRP1* |
| Proliferation of T lymphocytes | 9.2E-03 | 19 | *BSG, CD33, CD3G, CD6, DLG1, EOMES, EPHB1, ETS2, FN1 (includes EG:100005469), GAB2, HLA-DQA1, ICOSLG, IFNGR1, ITGAL, LAT, PAWR, PIK3CD, TNFRSF4, VDR* |
| T cell development | 9.6E-03 | 16 | *BSG, CD3G, CD6, CSK, EOMES, ETS2, GAS6, HLA-DQA1, ICOSLG, IFNGR1, IL5RA, ITGA4, ITGAL, LAT, PIK3CD, TNFRSF4* |
| Development of blood cells | 1.1E-02 | 20 | *BSG, CD3G, CD6, CSK, EOMES, ETS2, GAS6, HLA-DQA1, ICOSLG, IFNGR1, IL5RA, ITGA4, ITGAL, KIAA0101, LAT, PGF, PIK3CD, POU2AF1, SH2B3, TNFRSF4* |
| Differentiation of epithelial cells | 1.2E-02 | 10 | *F11R, FLG, FN1 (includes EG:100005469), HPS4 (includes EG:192232), LOR, MET, OCA2 (includes EG:18431), POU2F3, TYRP1, VDR* |
| Differentiation of cells | 1.7E-02 | 49 | *ACVR1, ADAM22, ADCYAP1 (includes EG:11516), ANXA6, BSG, CAND2, CD3G, CNTN2, DHCR7, EOMES, ETS2, F11R, FLG, FN1 (includes EG:100005469), GAB2, HLA-DQA1, HOXD3, HPS4 (includes EG:192232), ICOSLG, IFITM2, IFNGR1, IL5RA, ITGAL, LAT, LOR, MECOM, MET, NEUROD1, NMT1, OCA2 (includes EG:18431), ONECUT2, PBX1, PCSK2, PGF, PIK3CD, POU2AF1, POU2F3, PRTN3, PTGER2, RYK (includes EG:140585), SBF1, SH2B3, SH3PXD2B, TLR6, TNFRSF4, TYRP1, VDR, WASF2, WFIKKN2* |
| Differentiation of embryonic tissue | 2.9E-02 | 3 | *CUL7, ETS2, PGF* |
| Differentiation of keratinocytes | 3.3E-02 | 5 | *FLG, FN1 (includes EG:100005469), LOR, POU2F3, VDR* |
|  |  |  |  |
| *Cellular Function and Maintenance* | | | |
| Function of T lymphocytes | 6.8E-04 | 13 | *CD3G, CTSC, DLG1, HLA-C, ICOSLG, IFNGR1, ITGAL, LAT, PAWR, PIK3CD, POU2AF1, TNFRSF4, VDR* |
| Function of lymphocytes | 2.4E-03 | 14 | *CD3G, CTSC, DLG1, HLA-C, ICOSLG, IFNGR1, ITGAL, LAT, PAWR, PIK3CD, POU2AF1, RHBDF2, TNFRSF4, VDR* |
| Respiratory burst of neutrophils | 2.8E-03 | 4 | *ADCYAP1 (includes EG:11516), FN1 (includes EG:100005469), ITGA4, ITGAL* |
| Function of cytotoxic T cells | 4.2E-03 | 4 | *CTSC, HLA-C, IFNGR1, ITGAL* |
| Homeostasis of leukocytes | 4.6E-03 | 18 | *BSG, CD3G, CD6, CSK, EOMES, ETS2, GAS6, HLA-DQA1, ICOSLG, IFNGR1, IL5RA, ITGA4, ITGAL, LAT, MECOM, PGF, PIK3CD, TNFRSF4* |
| Function of Th2 cells | 7.8E-03 | 4 | *ICOSLG, PAWR, POU2AF1, TNFRSF4* |
| Lymphocyte homeostasis | 8.6E-03 | 17 | *BSG, CD3G, CD6, CSK, EOMES, ETS2, GAS6, HLA-DQA1, ICOSLG, IFNGR1, IL5RA, ITGA4, ITGAL, LAT, PGF, PIK3CD, TNFRSF4* |
| T cell development | 9.6E-03 | 16 | *BSG, CD3G, CD6, CSK, EOMES, ETS2, GAS6, HLA-DQA1, ICOSLG, IFNGR1, IL5RA, ITGA4, ITGAL, LAT, PIK3CD, TNFRSF4* |
| Function of leukocytes | 1.2E-02 | 18 | *CD3G, CTSC, DLG1, F11R, GAB2, HLA-C, ICOSLG, IFNGR1, ITGAL, LAT, PAWR, PIK3CD, POU2AF1, PTGER2, RHBDF2, TLR6, TNFRSF4, VDR* |
| Cellular homeostasis | 1.4E-02 | 36 | *ADCYAP1 (includes EG:11516), ANXA6, AQP2, ATG9A, ATP2A1, BSG, CD3G, CD6, CLSTN1, COX5A (includes EG:100537689), CSK, DAPK2, DLG1, EOMES, ETS2, FN1 (includes EG:100005469), GABARAPL1, GAS6, HLA-DQA1, ICOSLG, IFNGR1, IL5RA, ITGA4, ITGAL, LAT, MAP1LC3B, MECOM, MET, MTMR3, NEUROD1, PGF, PIK3CD, PTGER2, SCN8A, TNFRSF4, VDR* |
| Function of regulatory T lymphocytes | 1.7E-02 | 3 | *DLG1, LAT, TNFRSF4* |
| Function of cardiomyocytes | 1.8E-02 | 3 | *PNPLA2, TTN, VDR* |
| Engulfment of cells | 2.9E-02 | 10 | *APOH, CLEC4M, CORO1C, CSK, EPN2, GAB2, GAS6, ICOSLG, PRTN3, SIGLEC11* |
| Phagocytosis of cells | 3.1E-02 | 8 | *CLEC4M, CORO1C, CSK, GAB2, GAS6, ICOSLG, PRTN3, SIGLEC11* |
|  |  |  |  |
| *Cellular Growth and Proliferation* |  |  |  |
| Proliferation of immune cells | 1.8E-03 | 26 | *BSG, CD33, CD3G, CD6, DLG1, EOMES, EPHB1, ETS2, FN1 (includes EG:100005469), GAB2, HLA-DQA1, ICOSLG, IFNGR1, IL5RA, ITGAL, LAT, MLLT3, PAWR, PBX1, PIK3CD, POU2AF1, SH2B3, TNFRSF4, TYR, TYRP1, VDR* |
| Proliferation of blood cells | 1.9E-03 | 27 | *BSG, CD33, CD3G, CD6, DLG1, EOMES, EPHB1, ETS2, FN1 (includes EG:100005469), GAB2, HLA-DQA1, ICOSLG, IFNGR1, IL5RA, ITGAL, LAT, MECOM, MLLT3, PAWR, PBX1, PIK3CD, POU2AF1, SH2B3, TNFRSF4, TYR, TYRP1, VDR* |
| Proliferation of lymphocytes | 2.9E-03 | 24 | *BSG, CD33, CD3G, CD6, DLG1, EOMES, EPHB1, ETS2, FN1 (includes EG:100005469), GAB2, HLA-DQA1, ICOSLG, IFNGR1, IL5RA, ITGAL, LAT, PAWR, PIK3CD, POU2AF1, SH2B3, TNFRSF4, TYR, TYRP1, VDR* |
| Proliferation of T lymphocytes | 9.2E-03 | 19 | *BSG, CD33, CD3G, CD6, DLG1, EOMES, EPHB1, ETS2, FN1 (includes EG:100005469), GAB2, HLA-DQA1, ICOSLG, IFNGR1, ITGAL, LAT, PAWR, PIK3CD, TNFRSF4, VDR* |
|  |  |  |  |

**Table S11**. Enrichment for stratified SNPs at genetic loci known to be associated with respective phenotype in GWA studies. Observed: no of stratified SNPs (Fst>0.10) within 500kb of the reported sentinel SNPs. Predicted: mean no of SNPs expected to fall within 500kb of the sentinel SNPs under null hypothesis. Expectation based on permutation testing: 10,000 runs of SNP sets matched to the stratified SNPs based on allele frequency and gene proximity, but otherwise selected at random. Enrichment: observed / expected. P: exact probability of the observed based on the distribution of expected generated by permutation testing.

| **Phenotype** | **Observed** | **Predicted** | **Enrichment** | **P** |
| --- | --- | --- | --- | --- |
|  |  |  |  |  |
| Eye colour [1–3] | 593 | 48 | 12.23 | <1.0E-05 |
| Vitamin D [4] | 133 | 13 | 10.46 | 9.4E-03 |
| Freckling [2,3,5] | 382 | 34 | 11.32 | 7.0E-04 |
| Hair colour [2,3,5] | 327 | 49 | 6.64 | 3.4E-03 |
|  |  |  |  |  |
| Waist-hip ratio [6] | 300 | 87 | 3.45 | 1.9E-02 |
| Height [7] | 1498 | 1197 | 1.25 | 1.3E-01 |
| Body mass index [8] | 144 | 209 | 0.69 | 6.7E-01 |
|  |  |  |  |  |
| Resting HR [9] | 171 | 34 | 5.04 | 2.5E-02 |
| Diastolic BP [10] | 399 | 95 | 4.19 | 3.8E-03 |
| Systolic BP [10] | 328 | 108 | 3.03 | 2.0E-02 |
|  |  |  |  |  |
| Triglycerides [11] | 488 | 205 | 2.38 | 2.1E-02 |
| LDL cholesterol [11] | 573 | 226 | 2.53 | 1.3E-02 |
| HDL Cholesterol [11] | 443 | 304 | 1.46 | 1.5E-01 |
|  |  |  |  |  |
| Glucose [12,13] | 246 | 107 | 2.31 | 7.1E-02 |
| Insulin [13] | 44 | 35 | 1.27 | 2.5E-01 |
| Type-2 diabetes [14–18] | 280 | 186 | 1.51 | 1.7E-01 |
| Mean cell volume [19] | 892 | 280 | 3.19 | 6.0E-04 |
| Platelet count [20] | 1098 | 339 | 3.23 | 1.0E-04 |
| Haemoglobin [19] | 152 | 135 | 1.12 | 3.3E-01 |
|  |  |  |  |  |

**Table S12**. P values for enrichment of functional classes amongst autosomal SNPs across a range of allele frequencies.

| **Allele frequency >** | **0.01** | **0.02** | **0.05** | **0.10** | **0.20** |
| --- | --- | --- | --- | --- | --- |
|  |  |  |  |  |  |
| **South Asian specific SNPs** |  |  |  |  |  |
| 3PRIME_UTR | 1.0E+00 | 7.5E-01 | 9.3E-01 | 1.7E-02 | 1.7E-02 |
| 5PRIME_UTR | 4.9E-04 | 3.1E-03 | 2.1E-01 | 9.3E-01 | 1.0E+00 |
| DOWNSTREAM | 9.7E-10 | 7.7E-10 | 5.0E-04 | 8.8E-05 | 2.4E-01 |
| INTERGENIC | 7.9E-06 | 7.8E-01 | 6.2E-26 | 2.8E-35 | 7.1E-41 |
| INTRONIC | 8.8E-01 | 1.9E-06 | 1.0E-46 | 9.0E-50 | 1.4E-42 |
| NON_SYNONYMOUS_CODING | 7.0E-21 | 1.1E-11 | 2.3E-10 | 6.2E-11 | 1.7E-07 |
| SPLICE_SITE | 1.0E+00 | 8.5E-01 | 7.7E-01 | 4.9E-01 | 6.9E-01 |
| STOP_GAINED | 1.3E-01 | 5.5E-01 | 1.0E+00 | 1.0E+00 | 1.0E+00 |
| SYNONYMOUS_CODING | 8.3E-01 | 9.4E-01 | 4.5E-03 | 1.1E-04 | 2.1E-03 |
| UPSTREAM | 4.8E-24 | 2.2E-29 | 1.2E-20 | 4.8E-14 | 4.0E-05 |
|  |  |  |  |  |  |
| **SNPs shared with 1000G populations** |  |  |  |  |  |
| 3PRIME_UTR | 2.6E-26 | 2.7E-28 | 3.6E-33 | 1.1E-38 | 2.3E-36 |
| 5PRIME_UTR | 2.0E-45 | 1.5E-52 | 6.9E-48 | 5.2E-32 | 6.4E-16 |
| DOWNSTREAM | 9.6E-47 | 7.2E-50 | 2.8E-30 | 1.4E-20 | 1.5E-06 |
| INTERGENIC | 1.1E-54 | 8.4E-97 | 6.4E-92 | 4.2E-115 | 3.3E-149 |
| INTRONIC | 5.0E-162 | 1.1E-237 | 1.4E-182 | 1.6E-177 | 2.9E-158 |
| NON_SYNONYMOUS_CODING | 4.5E-180 | 7.6E-209 | 7.0E-212 | 1.6E-195 | 6.0E-134 |
| SPLICE_SITE | 8.4E-01 | 9.8E-01 | 1.1E-01 | 3.6E-01 | 6.0E-01 |
| STOP_GAINED | 1.7E-17 | 1.9E-15 | 9.6E-11 | 1.6E-08 | 1.1E-08 |
| SYNONYMOUS_CODING | 2.2E-04 | 7.4E-03 | 6.1E-04 | 8.2E-05 | 6.7E-04 |
| UPSTREAM | 1.7E-138 | 8.8E-154 | 3.8E-108 | 2.2E-69 | 4.7E-20 |
|  |  |  |  |  |  |

**Table S13**. PCR primers for validation of indel calling by Sanger sequencing.

| **Chr** | **Position** | **Forward primer** | **Reverse primer** |
| --- | --- | --- | --- |
| 1 | 34421405 | AGGGTTGTGGTGAGGATTGA | CGAGGTGGAGATCAGCCTAC |
| 1 | 39071780 | CAAGGTCTCTTGGAGGCTGA | TCCATTGATGAGCATCTCTCC |
| 1 | 57227460 | GTATGCCCAATGCCAAGTTC | GTGTTGGACCAATTGCCATA |
| 1 | 63570931 | GCATTGGACTGAACATTTGG | AAAGGCTGTGCATGGGTACT |
| 1 | 173237052 | CAGCCAGGGCTACAGTCATT | TGAGAAATCTGCTGTCATTTGC |
| 1 | 229302642 | AAAGGAGCCCTCTAGCCTGT | GAGGCTAGCATTGCCCAGTA |
| 2 | 70513648 | AATGGCCTCAATGGATCACT | GCCTCCTGTTCCATCTCTGA |
| 2 | 107231589 | GGTATCTGAGGTGGCAGGAA | CTCTGGTGGGGAGACACAGT |
| 2 | 239331251 | GATAAATGCTGCTGGGAGGA | GGGCTCACAGACTCCTTCAC |
| 3 | 10434045 | TGAACTTTCCAGGGTGGGTA | GCGCTTTGACTTGGATGTCT |
| 3 | 24863547 | TGCTTGAAAGGGTTTTCCTG | CACACATCTGCAGCACTGAC |
| 3 | 51385959 | TCAGACCTACTGCCAAGTCCT | CGTGTGGCTTTCTAGCTCAA |
| 3 | 109344848 | CTAGGCAAAGCGTCAGATCC | AAATCCTTCACATACAGGGCTTA |
| 3 | 193964511 | AATAGGCTTAGGGGGCTCTG | ACGTTGATTAAATTAGACAAATGTCAG |
| 4 | 6388911 | TCCTCAAAGACCTGTTGCAT | GGGTGCCATTATTTCTGCTG |
| 5 | 71035744 | CTGGAGGCAACCACTATTCC | GCGAAAGAATGGACTCTAATAGC |
| 5 | 163198560 | GGACGAGGACCTTGCTACAG | CCATCCCACTCACTCCAACT |
| 5 | 163198560 | CAGGAAGCTGAGATGGGAGA | GCAGTCAGTGGTGACTATTCAAG |
| 6 | 85759289 | CTCTGTCCAAACCCCTGAAA | AAGAGCCATTTTGTCAGAATCC |
| 7 | 155599630 | GCACGTCCTCTCAGGGTAGA | GCCTTCAATTCTCCAAGCAG |
| 10 | 90335467 | CAAAACCACCCAAAAAGCAG | AAGGCAGGGCCAGGTTATAC |
| 11 | 10725946 | GCAGAGGTGGCAGTGAGTTA | GGGGCAGAATGCAGAATGCAGAAAGTA |
| 12 | 18570680 | CATCAACATGGACCGAACAC | GGCAATGAAAGAGAGGGATG |
| 12 | 20851211 | AAACTTACTTCAACATTCGATGC | TGGATTCCTCTAGGGAGTGG |
| 13 | 24690876 | CTGCCTTGCACTCATCTCCT | AAGGCCCTTTCTCCAAACAT |
| 13 | 63883368 | GGGGAGTTCTAGGGTGATGA | AACCTGGGAGGTGGAGATTC |
| 14 | 40301837 | TATCCCCGGTGGAAACAGTA | TGGAAGAATTGACTAGGGTGCT |
| 14 | 40657690 | CCCTGGAATGTGGCTAGTGT | GGGAAAAACATAAGCTATGCAA |
| 17 | 20200129 | GAGGTCCTTCACATCCCTTG | GTGTGGAGCAGAGCACAGAA |
| 17 | 26001819 | GCACCCAGTTCCTTGTCACT | CTCCTGGAATTTGGCATCAC |
| 18 | 21375698 | CCTTACAATTTCACGTGACAGAA | CCTATCAGATCAGGGCTCCA |
| 18 | 43724710 | CCTGGCCACATTCCTTTTAT | TCCCATATTGCTGGGATTGT |
| 18 | 76987962 | AGCCCAGCACTTGTTTCTGT | TGTCCTGCATTGATTCACTC |
| 19 | 8221572 | GGCTGGAGTGCGGTAGTAT | GGGCCACCTAGTGTGTGATT |
| 22 | 40405215 | GCTTCAGATGTGGGGAGTTC | GGCCAGAGAAAGACAACACC |

Reference List

1. Liu F, Wollstein A, Hysi PG, Ankra-Badu GA, Spector TD, et al. (2010) Digital quantification of human eye color highlights genetic association of three new loci. PLoS Genet 6: e1000934.

2. Sulem P, Gudbjartsson DF, Stacey SN, Helgason A, Rafnar T, et al. (2008) Two newly identified genetic determinants of pigmentation in Europeans. Nat Genet 40: 835-837.

3. Eriksson N, Macpherson JM, Tung JY, Hon LS, Naughton B, et al. (2010) Web-based, participant-driven studies yield novel genetic associations for common traits. PLoS Genet 6: e1000993.

4. Ahn J, Yu K, Stolzenberg-Solomon R, Simon KC, McCullough ML, et al. (2010) Genome-wide association study of circulating vitamin D levels. Hum Mol Genet 19: 2739-2745.

5. Sulem P, Gudbjartsson DF, Stacey SN, Helgason A, Rafnar T, et al. (2007) Genetic determinants of hair, eye and skin pigmentation in Europeans. Nat Genet 39: 1443-1452.

6. Heid IM, Jackson AU, Randall JC, Winkler TW, Qi L, et al. (2010) Meta-analysis identifies 13 new loci associated with waist-hip ratio and reveals sexual dimorphism in the genetic basis of fat distribution. Nat Genet 42: 949-960.

7. Lango AH, Estrada K, Lettre G, Berndt SI, Weedon MN, et al. (2010) Hundreds of variants clustered in genomic loci and biological pathways affect human height. Nature 467: 832-838.

8. Speliotes EK, Willer CJ, Berndt SI, Monda KL, Thorleifsson G, et al. (2010) Association analyses of 249,796 individuals reveal 18 new loci associated with body mass index. Nat Genet 42: 937-948.

9. Eijgelsheim M, Newton-Cheh C, Sotoodehnia N, de Bakker PI, Muller M, et al. (2010) Genome-wide association analysis identifies multiple loci related to resting heart rate. Hum Mol Genet 19: 3885-3894.

10. Ehret GB, Munroe PB, Rice KM, Bochud M, Johnson AD, et al. (2011) Genetic variants in novel pathways influence blood pressure and cardiovascular disease risk. Nature 478: 103-109.

11. Teslovich TM, Musunuru K, Smith AV, Edmondson AC, Stylianou IM, et al. (2010) Biological, clinical and population relevance of 95 loci for blood lipids. Nature 466: 707-713.

12. Dupuis J, Langenberg C, Prokopenko I, Saxena R, Soranzo N, et al. (2010) New genetic loci implicated in fasting glucose homeostasis and their impact on type 2 diabetes risk. Nat Genet 42: 105-116.

13. Manning AK, Hivert MF, Scott RA, Grimsby JL, Bouatia-Naji N, et al. (2012) A genome-wide approach accounting for body mass index identifies genetic variants influencing fasting glycemic traits and insulin resistance. Nat Genet 44: 659-669.

14. Voight BF, Scott LJ, Steinthorsdottir V, Morris AP, Dina C, et al. (2010) Twelve type 2 diabetes susceptibility loci identified through large-scale association analysis. Nat Genet 42: 579-589.

15. Perry JR, Voight BF, Yengo L, Amin N, Dupuis J, et al. (2012) Stratifying type 2 diabetes cases by BMI identifies genetic risk variants in LAMA1 and enrichment for risk variants in lean compared to obese cases. PLoS Genet 8: e1002741.

16. Zeggini E, Scott LJ, Saxena R, Voight BF, Marchini JL, et al. (2008) Meta-analysis of genome-wide association data and large-scale replication identifies additional susceptibility loci for type 2 diabetes. Nat Genet 40: 638-645.

17. Scott LJ, Mohlke KL, Bonnycastle LL, Willer CJ, Li Y, et al. (2007) A genome-wide association study of type 2 diabetes in Finns detects multiple susceptibility variants. Science 316: 1341-1345.

18. Qi L, Cornelis MC, Kraft P, Stanya KJ, Linda Kao WH, et al. (2010) Genetic variants at 2q24 are associated with susceptibility to type 2 diabetes. Hum Mol Genet 19: 2706-2715.

19. Van der Harst P, Zhang W, Mateo L, I, Rendon A, Verweij N, et al. (2012) Seventy-five genetic loci influencing the human red blood cell. Nature 492: 369-375.

20. Gieger C, Radhakrishnan A, Cvejic A, Tang W, Porcu E, et al. (2011) New gene functions in megakaryopoiesis and platelet formation. Nature 480: 201-208.
